# Ferroelectric brightening of spin‑forbidden dark excitons in a WSe_2_/hybrid-perovskite heterostructure

**DOI:** 10.1038/s41467-026-72143-y

**Published:** 2026-05-09

**Authors:** Xinyun Wang, Magdalena Grzeszczyk, Maxim Trushin, Ivan Verzhbitskiy, Dmitrii Litvinov, Yi Wei Ho, Yuan Chen, Zhenyue Wu, Mykola Telychko, Chuanqi Zhang, Andres Granados del Aguila, Kuan Eng Johnson Goh, Xinwei Li, Goki Eda, Shaffique Adam, Maciej Koperski, Kian Ping Loh

**Affiliations:** 1 Department of Chemistry, National University of Singapore, Singapore, Singapore; 2 Department of Physics, National University of Singapore, Singapore, Singapore; 3 Institute for Functional Intelligent Materials, National University of Singapore, Singapore, Singapore; 4 Centre for Advanced 2D Materials and Graphene Research Centre, National University of Singapore, Singapore, Singapore; 5 Department of Materials Science Engineering, National University of Singapore, Singapore, Singapore; 6 Quantum Innovation Center, Agency for Science Technology and Research, Singapore, Singapore; 7 Institute of Materials Research and Engineering, Agency for Science Technology and Research, Singapore, Singapore; 8 Division of Physics and Applied Physics, School of Physical and Mathematical Sciences, Nanyang Technological University, Singapore, Singapore

**Keywords:** Two-dimensional materials, Two-dimensional materials, Quantum optics

## Abstract

Long-lived dark excitons in monolayer WSe_2_ present promising candidates for carrying spin and valley information, but their optical access and spin manipulation have conventionally required the use of strong external magnetic fields. Here, using a ferroelectric hybrid perovskite heterostructure, we leverage the ferroelectric proximity effect to break the WSe_2_’s in-plane rotational symmetry and brighten the spin-forbidden dark excitons under zero magnetic field conditions. Furthermore, we show that the twist angle between the WSe_2_ and perovskite crystals controls the ferroelectric coupling strength and valley-contrasting polarization. Our proposed mechanism, supported by a four-band tight-binding model, suggests that the ferroelectric proximity effect induces an asymmetric intersublattice interaction, generating an effective in-plane spin-orbit coupling (SOC) field that rotates spin/valley polarization and brightens dark excitons. Our work establishes ferroelectric proximity coupling as a symmetry-tunable, magnetic-field-free strategy for spin exciton control in two-dimensional semiconductors.

## Abstract (web summary)

The authors leverage the ferroelectric proximity effect of a ferroelectric hybrid perovskite heterostructure to break the in-plane rotational symmetry of WSe2 and brighten the spin-forbidden dark excitons under zero magnetic field conditions.

## Introduction

In monolayer WSe_2_, the band-edge exciton is theoretically predicted and experimentally confirmed to be spin-forbidden and optically dark. Due to short-range exchange interactions, this dark exciton manifold splits into two distinct states: a strictly dark exciton (X^D^) and a gray exciton (X^G^)^1–4^. These states form as coherent superpositions of valley-polarized configurations, where X^D^ is antisymmetric and completely dipole-forbidden, remaining optically inactive under out-of-plane detection, while X^G^ is symmetric and possesses a small out-of-plane dipole moment, granting it limited but nonzero oscillator strength^5,6^. Because of their suppressed radiative decay, these long-lived dark and gray excitons serve as protected reservoirs for valley and spin information. Their optical access and spin state control, enabled via magnetic fields or tailored optical pulses, make monolayer WSe_2_ a highly promising material for valleytronics and quantum information processing.

Dark excitons have been activated most directly by in‑plane magnetic fields that mix bright and dark states, revealing strong emissions and long valley lifetimes^7–9^. Magnetic‑free routes, such as near‑field plasmonic coupling^10–13^ and tip‑enhanced nano‑cavity control^14^, preferentially intensify gray‑exciton emission by coupling to its out‑of‑plane dipole moment and increasing the radiative rate through strong Purcell enhancement. In this context, electric-field-assisted SOC routes are appealing in that they provide an intrinsic approach to symmetry breaking and spin mixing without an external magnetic field and can be combined with optical cavities for further enhancement. However, conventional Rashba SOC induced by an out-of-plane electric field is intrinsically weak, owing to the small Rashba coefficient around band extrema. As a result, appreciable spin mixing requires either very large local electric fields or alternative symmetry-breaking mechanisms^15–17^.

Here, we demonstrate the magnetic-free brightening of dark exciton (X^D^) in monolayer WSe_2_, enabled by ferroelectric proximity and its intrinsic Kane-Mele (KM) SOC, realized by interfacing WSe_2_ with in-plane ferroelectric hybrid perovskite (TMA)_3_Sb_2_Cl_9_ (TSC). Both the ferroelectric brightening and the polarization-phase difference of the dark/gray exciton doublet are tunable with the interlayer twist angle. Our theoretical analysis indicates that the directional in-plane ferroelectric field couples to the WSe_2_ electronic wavefunctions and generates a sublattice-asymmetric spin-orbit interaction, producing an effective in-plane SOC field that rotates the exciton spin configuration and activates the otherwise spin-forbidden transition. This mechanism is supported by the observed symmetry reduction, the twist-angle-dependent magneto-photoluminescence (PL) response, and the polarization phase shift of the dark/gray exciton doublet. Together, these results establish ferroelectric proximity as a symmetry-tunable, magnetic-free route to static spin and exciton control in two-dimensional quantum materials.

## Results

**Fig. 1:**
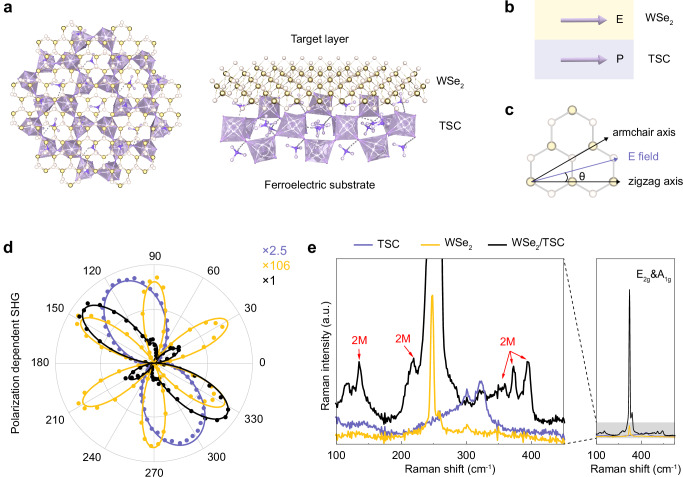
Symmetry breaking in WSe_2_ via ferroelectric proximity coupling. **a** Schematic illustration of the heterostructure: monolayer WSe_2_ on a 2D ferroelectric perovskite (TSC). **b** The ferroelectric polarization $P$ imprints a microscopic in‑plane electric field $E$ across the interface. **c** The twist angle ${\theta }$ is defined between the WSe_2_ zigzag axis and the TSC ferroelectric polarization. **d** Polar plot of co-polarized SHG response from TSC (purple), monolayer WSe_2_ (yellow), and the WSe_2_/TSC heterostructure (black) at a twist angle ${\theta }$ of 6.7°. Symbols denote experimental data, and solid lines are fits. The SHG intensities are scaled by the factors indicated in the panel for clarity. The monolayer WSe_2_ shows a six‑lobe polar pattern, whereas the WSe_2_/TSC heterostructure exhibits a strongly asymmetric pattern that cannot be achieved by linear superposition of the individual crystal tensors. Fits require ${C}_{1}$ symmetry for WSe_2_, indicating the three-fold rotational and horizontal mirror‑symmetry breaking (see Supplementary Section 1 for fitting d**e**tails). **e** Raman scattering spectra from the same regions. Additional weak features labeled ‘2 M’ correspond to second‑order zone‑edge phonon combinations that become visible when selection rules are relaxed by interfacial symmetry breaking (see Table S1).

Ferroelectric proximity coupling is implemented in a vertical heterostructure (Fig. 1a; fabrication details in “Methods”). The in-plane ferroelectric polarization in TSC produces bound surface charges and an associated electrostatic potential that extends into the adjacent WSe_2_ layer, effectively “imprinting” a directional in-plane electric field onto WSe_2_ (Fig. 1b). The motivation for choosing TSC as the ferroelectric substrate is twofold: (1) Its dangling-bond-free surface forms atomically clean van der Waals interface with WSe_2_, enabling high sample quality and efficient proximity field transfer without chemical hybridization; (2) Its large bandgap makes it an optically transparent substrate and avoids interfacial charge transfer during light excitation. Detailed characterization of TSC has been reported in our previous work^18^ and Supplementary Fig. S6. Importantly, the interlayer twist angle offers an additional control knob, allowing the proximity field to align preferentially with either the zigzag or armchair directions of WSe_2_ (Fig. 1c).

Linearly co-polarized second-harmonic generation (SHG) measurement is first used to determine the stacking angle of the heterostructure by examining the crystallographic orientation of individual layers. In accordance with previous reports, monolayer WSe_2_ exhibits six symmetric lobes (Fig. 1d) under normal incidence, reflecting its three-fold rotational symmetry (${D}_{3h}$ point group)^19,20^. The TSC crystal shows a two-lobe polarization pattern, corresponding to its ${C}_{s}$ symmetry. After fitting the SHG polar map from individual layers, an interfacial twist-angle of 6.7° is determined for this heterostructure.

Notably, in this heterostructure region, the obtained SHG polar map cannot be fitted using any linear superposition of the respective susceptibility tensor of ${D}_{3h}$ and ${C}_{s}$ symmetry point group. We further tested each subgroup under the ${D}_{3h}$ symmetry and found that only the lowest ${C}_{1}$ point group can fit the experimental data. Due to Neumann’s principle, the crystal symmetry is encoded in the second-order nonlinear susceptibility tensor ${{{{\rm{\chi }}}}}^{(2)}$, determining the efficiency and occurrence of the SHG process. Therefore, the reduced ${{{{\rm{\chi }}}}}^{(2)}$ tensor indicates the broken three-fold rotational and horizontal mirror reflection symmetry of WSe_2_ in the heterostructure. A detailed SHG analysis is described in Supplementary Section 1.

Furthermore, the Raman spectrum of the heterostructure (Fig. 1e) exhibits several additional weak bands (2 M) around ~150, ~220, and ~350-400 cm^−1^. These features are commonly assigned to double‑resonant multi‑phonon Raman processes involving zone‑edge (M/K) phonons^21–24^ (Supplementary Table S1). In a perfect crystal, first‑order Raman scattering is dominated by Γ‑point phonons ($q\approx 0$), so finite‑momentum (M/K) contributions are typically weak. However, they could be observed when momentum/selection rules are relaxed by interfacial symmetry lowering, strain or disorder, consistent with our SHG evidence for reduced symmetry. Similar 2 M bands are also observed when WSe_2_ is interfaced with other in‑plane ferroelectrics (Supplementary Fig. S7), supporting that the symmetry reduction is imprinted onti WSe_2_ rather than originating from substrate vibrational modes.

**Fig. 2:**
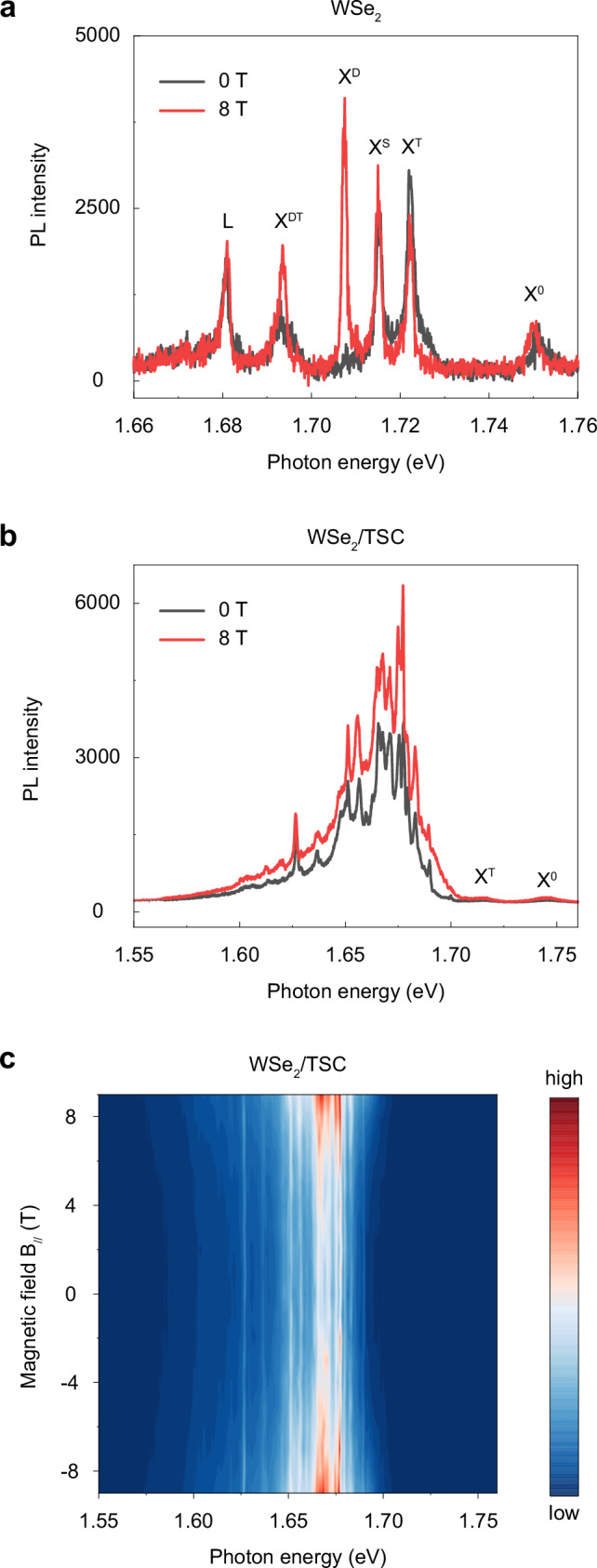
Observation of spin-forbidden dark excitons in WSe_2_/TSC heterostructures at zero magnetic field. **a** PL spectra of a pristine WSe_2_ monolayer at 0 T and 8 T. **b** False‑color map of magneto-PL from the 6.7° stacked WSe_2_/TSC heterostructure. **c** PL spectra of the heterostructure at 0 T and 8 T. All magneto-PL spectra were acquired at 1.6 K, unless otherwise specified.

We first investigate a pristine WSe_2_ monolayer as a benchmark for identifying spin‑forbidden emission (Fig. 2a). At zero magnetic field, the PL is dominated by the bright neutral exciton X^0^ at 1.750 eV, accompanied by nearby charged‑exciton resonances (X^T^ at 1.722 eV and X^S^ at 1.715 eV) and an occasional lower‑energy localized‑exciton line (L). Upon applying an in‑plane magnetic field (B_//_) in the Voigt geometry, a distinct low‑energy feature (X^D^) at 1.707 eV becomes clearly visible and increases in intensity. A further red‑shifted peak at 1.693 eV is assigned to the corresponding dark trion X^DT^, based on its magneto response and characteristic energy offset relative to X^D^. As demonstrated in previous studies^3,7–9^, the application of B_//_ can rotate the spin polarization through interband wavefunction mixing and thereby brighten spin‑forbidden dark excitonic states. Because B_//_ is transverse to the Ising‑like (z-polarized) spin axis in TMDs, this Voigt‑geometry brightening provides a particularly discriminating probe of spin selection‑rule breaking.

In contrast to the monolayer, a new group of low‑energy peaks emerges at zero field and becomes further enhanced under B_//_ field (Fig. 2b, c) in the WSe_2_/TSC heterostructure, consistent with a spin‑forbidden origin. Differential‑reflectance measurements confirm that TSC is optically transparent in the relevant spectral window and that the heterostructure response is dominated by the WSe_2_ A‑exciton resonance (Supplementary Fig. S8), indicating that the emergent PL features are associated with WSe_2_ rather than the substrate. The rich array of emergent excitonic features is likely attributable to proximity-induced band splitting and phonon-assisted processes. A detailed microscopic assignment of each peak is beyond the scope of this work, hence we focus on elucidating the dark nature and brightening mechanism of the dominant peaks.

The dark nature is further corroborated by the g-factor extracted from out-of-plane magnetic-field (B_⊥_) dependent PL measurements in a valley-resolved configuration^25–29^ (see Supplementary Fig. S9). While the WS_2_/TSC heterostructure exhibits similar emergent excitonic peaks, no such counterpart excitonic states are observed in the MoSe_2_/TSC heterostructure (Supplementary Fig. S10). This material selectivity can be explained by the fact that the lowest exciton is exclusively dark in tungsten-based TMDs^30–33^. To further exclude alternative assignments, we performed comprehensive gate‑, power‑, and magnetic‑field‑dependent measurements (Supplementary Figs. S11–13). The emergent peaks maximize near the charge‑neutrality region and follow the neutral‑exciton gating trend, which is inconsistent with trions or other charged species. Many-body states can be excluded because their integrated intensity exhibits an approximately linear power dependence, indicative of a single-exciton transition rather than a biexciton/multiexciton complex. Finally, defect-bound emitters are ruled out because the peaks display the characteristic Voigt‑geometry magnetic brightening expected for spin‑forbidden excitons. In contrast, a defect-localized emitter measured under identical conditions shows no such brightening. Collectively, this evidence robustly identifies the emergent peaks as neutral, spin-forbidden excitons that are brightened via the ferroelectric proximity effect.

**Fig. 3:**
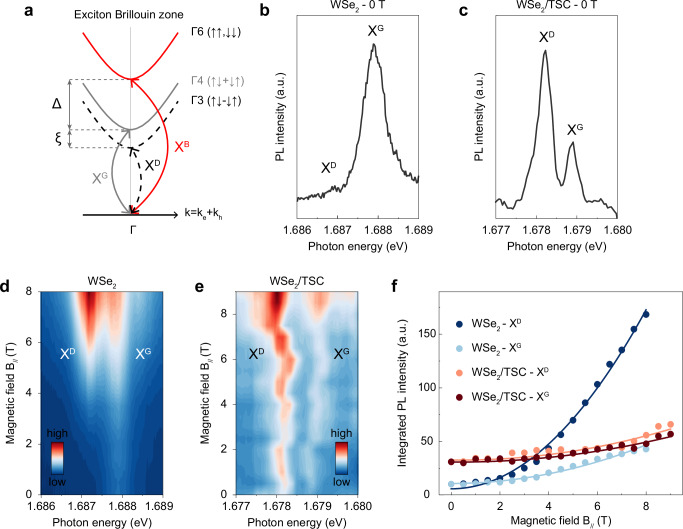
Magnetophotoluminescence signatures of dark-gray exciton doublet. **a** Exciton fine structure of a pristine WSe_2_ monolayer (point group ${D}_{3h}$). The bands are labeled with the irreducible representations of the ${D}_{3h}$ point group. $\Delta$ is the spin splitting from on-site SOC, and $\xi$ is the fine splitting from the short-range exchange interaction. Antiparallel spins at the band edges render the lowest neutral exciton X^D^ optically dark, while the higher energy state X^G^ acquires a finite out‑of‑plane dipole moment and is optically accessible. Irreducible‑representation la**b**els follow ${D}_{3h}$. **b**, **c** High-resolution PL spectra of a monolayer and a 6.7° stacked heterostructure at zero magnetic field. **d**, **e** Corresponding magneto‑PL map (in‑plane magnetic field B_//_). **f** Integrated intensities of X^D^/X^G^ doublets as a function of B_//_ field. The parabolic functions ($I={I}_{0}+\alpha {B}_{\parallel }^{2}$) are employed for the fitting. The reduced curvature $\alpha$ in the heterostructure indicates weaker magnetic‑field‑induced spin mixing, consistent with static zero‑field brightening induced by ferroelectric proximity.

Next, we turn to the fine structure of the dark manifold and examine whether the newly observed peaks are associated with the gray exciton state. Figure 3a illustrates the exciton fine structure of a pristine WSe_2_ monolayer. In monolayer WSe_2_, this doublet can be resolved under an external in-plane magnetic field at higher energy resolution, exhibiting a splitting energy of ~663 μeV^3,5^ (Fig. 3b). At zero magnetic field, X^D^ is absent, while X^G^ may be observed in high-quality samples, characterized by in-plane emission direction and attributed to the large numerical aperture of the objective lens. In this 6.7° stacked heterostructure, the doublet fine structures can also be resolved for each dark state. Figure 3c highlights the dominant exciton doublet at 1.678–1.679 eV. Importantly, both X^D^ and X^G^ exhibit intensive emission at zero magnetic field.

Similar to the magneto-response of the pristine monolayer, the exciton doublet in heterostructure shows further enhancement under applied B_//_ fields, confirming their spin-forbidden dark nature (Fig. 3d, e). The spectral wandering of the doublet can be attributed to fluctuations of the ferroelectric electric field, giving rise to a jittering effect (Supplementary Fig. S14). Furthermore, in both systems, the emission intensities of the X^G^ and X^D^ excitons increase parabolically with the in-plane magnetic field, following $I={I}_{0}+\alpha {B}_{\parallel }^{2}$ (ref. ^3,4^; Fig. 3f). The smaller proportionality coefficient ($\alpha$) in the heterostructure reflects reduced magnetic-field-induced spin mixing compared to the pristine monolayer, consistent with static zero‑field brightening driven by ferroelectric proximity.

**Fig. 4:**
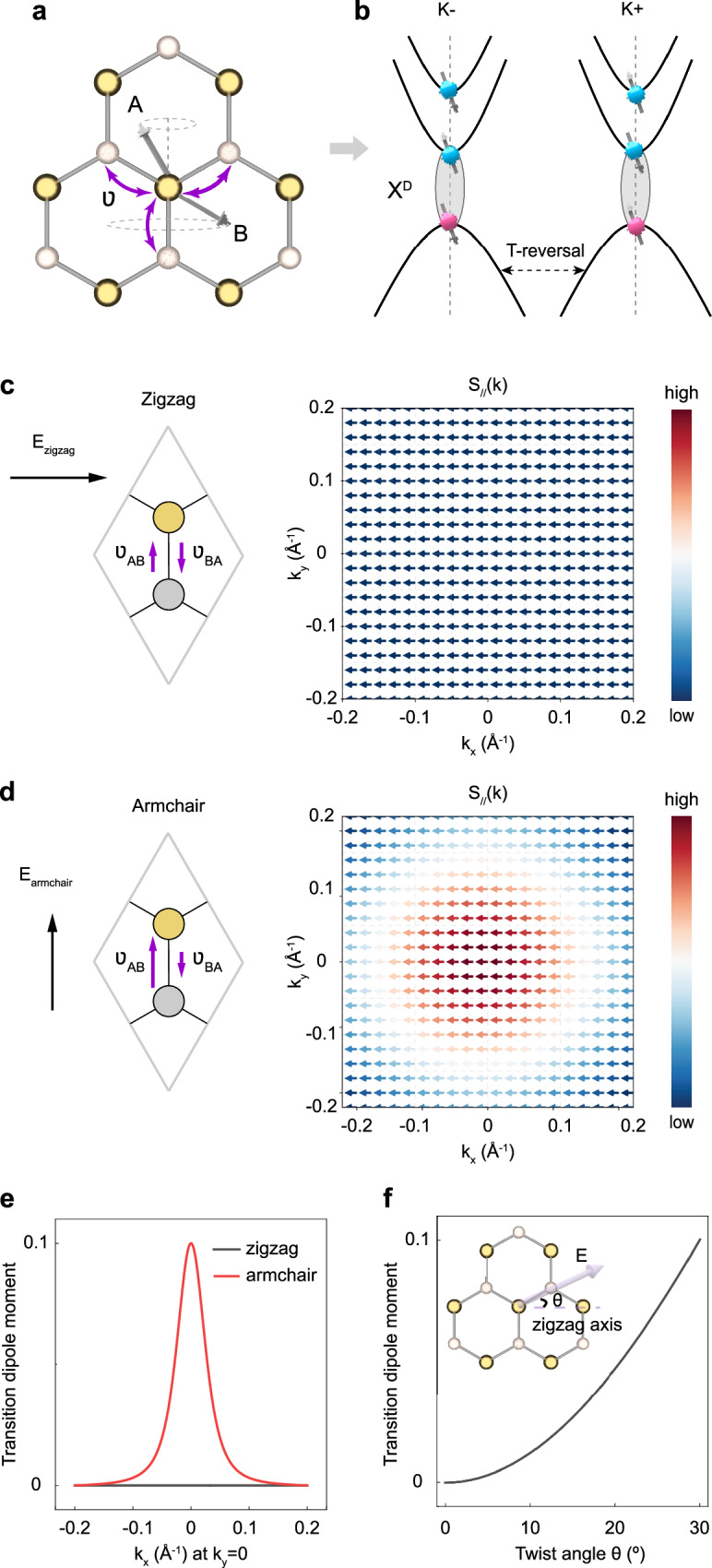
Theoretical investigation of spin canting induced by asymmetric intersublattice interaction. **a** Schematic diagram of the spin-orbit-coupled honeycomb lattice with resolved sublattices. The spin reorientation arises from KM-type SOC modified by the asymmetrical hopping between the two sublattice sites. **b** Schematic illustrates the spin canting at band edges. **c**, **d** Calculated quiver maps of in-plane spin components at the lowest conduction band of WSe_2_ in zigzag- and armchair-aligned heterostructure. Arrows indicate spin orientation, and color encodes magnitude. **e** Calculated $k$-dependent transition dipole moment. **f** Calculated twist-angle-dependent transition dipole moment at $k=0$. The inset illustrates the twist angle (${\theta }$) between the zigzag axis of WSe_2_ and the in-plane ferroelectric field.

We now propose a mechanism for dark-exciton brightening based on the ferroelectric proximity coupling. In honeycomb lattices, KM-type SOC couples real spin to the sublattice pseudospin and establishes an Ising-like spin texture at the band edges^34–36^. As a result, the spin orientation and the associated optical selection rules are highly sensitive to perturbations that differentiate the two sublattices (occupied by W and Se atoms in WSe_2_) and modify inter-sublattice interaction^37–39^. In the WSe_2_/TSC heterostructure, the in-plane ferroelectric polarization of TSC generates a directional interfacial electric field ($E$) onto the WSe_2_ lattice. This field lowers WSe_2_ symmetry and modifies the intersublattice electrostatic potential, leading to a SOC-enabled spin rotation (Fig. 4a). In a tight-binding picture, this imposes an asymmetry between nearest-neighbor A → B and B → A hopping amplitudes (${v}_{{AB}}{\ne v}_{{BA}}$). At the band edge, this hopping asymmetry could manifest as an effective in-plane SOC field, which reorients the band-edge spins and relaxes the spin-selection rule (Fig. 4b).

Next, we develop a four-band Hamiltonian to capture the ferroelectric proximity effect, in which the $E$ field enters as a direction-dependent perturbation term and determines the effective intersublattice hopping asymmetry. In particular, we focus on two representative alignment cases: the zigzag-aligned and armchair-aligned configurations, where the $E$ field is perpendicular and parallel to the WSe_2_ lattice, respectively, as illustrated in Fig. 4c, d. All other alignment configurations can be interpreted as linear combinations of these two cases. Notably, for the zigzag-aligned case, the net effective field along the lattice bonds is identical due to the intrinsic ${C}_{3}$ symmetry and the nearest-neighbor intersublattice hoppings yield symmetric amplitudes (${v}_{{AB}}\,=\,{v}_{{BA}}$), thus the effective in-plane SOC field is negligible (Fig. 4c). In contrast, for the armchair-aligned case, the intersublattice electrostatic potential is altered and the hopping strength becomes asymmetric (${v}_{{AB}}\ne {v}_{{BA}}$), giving rise to an in-plane effective SOC field and produces a pronounced local maximum at $k=0$ (Fig. 4d). Using the measured spontaneous polarization of around 2 µC/cm^2^, we estimate an unscreened maximum magnitude for the interfacial electric field of $E$ ~ 10^9 ^V/m. This gives rise to a spin‑orbit hopping energy ${E}_{{soc}}\,$~ 0.4 eV, which is sufficiently large to induce appreciable spin mixing. Detailed calculations are presented in Supplementary Section 2. The corresponding in-plane spin textures of the topmost valence band are shown in Supplementary Fig. S15.

Figure 4e displays the calculated $k$-dependent transition dipole moment, which vanishes for zigzag alignment but shows a pronounced increase for armchair alignment. This result is consistent with our experimental observations in multiple devices tested: the magneto-response is absent only in the zigzag-aligned heterostructure but appears in all other alignment configurations. The twist-angle dependent transition dipole moment (Fig. 4f) reveals a more specific correlation to the ferroelectric field projection. The monotonic dependence reveals that the spin rotation is proportional to the $E$ field projection along the armchair axis. In addition, we found that the transition dipole moment is independent of the magnitude of the on-site spin-flip term and is only determined by the hopping asymmetry, which is not considered by previous studies^6,15^. In fact, a finite on-site spin-flip term produces a uniform in-plane spin texture for both the lowest conduction band and highest valence band, with equally uniform magnitude but opposite sign. Consequently, the spins remain antiparallel, leaving the interband transition spin-forbidden.

**Fig. 5:**
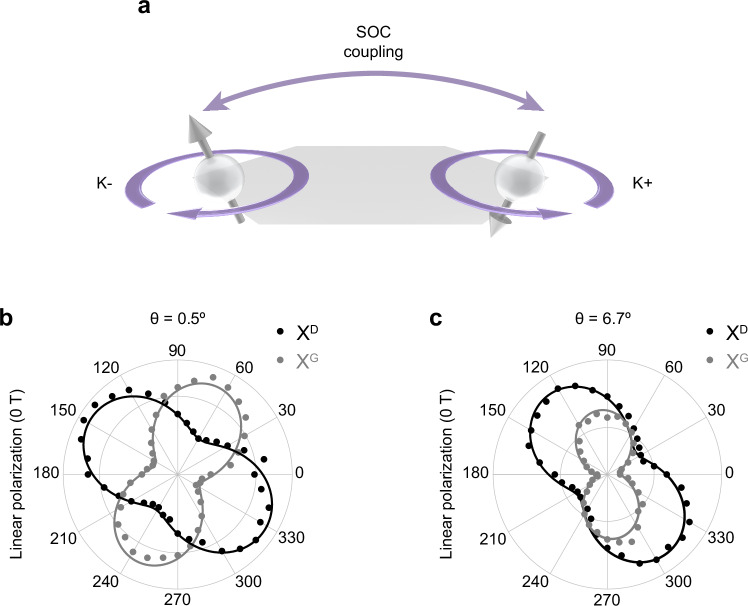
Twist-angle dependent phase difference. **a** Schematic of SOC‑induced valley mixing. Oppositely polarized spins couple to the time‑reversal‑related ${K}^{\pm }$ valleys, in which the valley polarization is encoded in the photon helicity (purple arrows). **b**, **c** Linear polarization-resolved emission of the dark-gray exciton doublet for heterostructures with a twist angle of 0.5° and 6.7° at 0 T.

The ferroelectric proximity mechanism is further supported by a reduction in the dark/gray exciton polarization phase difference. Unlike PL intensity, which can vary with extrinsic factors such as carrier doping and defects, the polarization orientation is an intrinsic property, arising from the coherent superposition of the two valley exciton states (Fig. 5a), and it therefore directly tracks the degree of intervalley coherence (valley mixing)^5,6,40^. In pristine monolayers, the doublet polarization exhibits a 90° phase difference, reflecting two orthogonal valley‑coherent superpositions^3,40,41^. In the heterostructures (Fig. 5b, c), this phase difference is reduced from 90° to 82.1° and 30.5° for twist angles of 0.5° and 6.7°, respectively, indicating a rotation of the valley-coherent basis. This observation follows naturally from the coupling between valley pseudospin and sublattice pseudospin: near$\,{K}^{-}$ and ${K}^{+}$, the in‑plane sublattice pseudospin texture carries opposite phase winding and encodes opposite valley chirality. Consequently, any perturbation that distinguishes A from B (A and B denote the two inequivalent basis sites within one unit cell, which is the sublattice (pseudospin) degree of freedom) introduces an effective intervalley-coupling term in the valley-coherent subspace, mixing the two valley components and rotating the polarization basis of the dark/gray doublet.

The observed phase reduction thus provides a robust fingerprint of our tight-binding theoretical model above, where the effective SOC field as well as the related spin and valley mixing degree is twist-angle dependent, vanishing at zigzag-aligned configuration and maximized at armchair-aligned configuration. It can be further supported by the twist-angle dependent magneto‑brightening curvature α due to a static zero‑field brightening effect (Supplementary Fig. S16). Although a systematic twist-angle dependence is limited by the demand for high-quality samples to resolve dark manifolds, the two representative heterostructures studied here are sufficient to distinguish the expected angular trend, following our calculated phase diagram based on the group theory (Supplementary Section 2.3).

In summary, we demonstrate the modulation of exciton spin and valley polarization via an interfacial ferroelectric proximity effect. This ferroelectric proximity is achieved in a van der Waals heterostructure combining a ferroelectric perovskite substrate with a monolayer of WSe_2_. At zero magnetic field, we observe the brightening of spin-forbidden dark-gray excitons in WSe_2_, as confirmed by their distinct magneto-optical response. We explain this effect using the KM-type SOC model, where the intersublattice hopping strength is regulated by the orientation of the ferroelectric field. This mechanism is additionally supported by the magnetic response and polarization properties of the dark/gray exciton doublet. Overall, our findings establish ferroelectric proximity coupling as an effective, magnetic-field-free strategy for manipulating spin and pseudospin degrees of freedom, an approach readily extendable to other low-dimensional quantum systems.

## Methods

### Sample fabrication

Because of the instability of TSC perovskite in air, an all-dry transfer method is used to fabricate the devices in a glove box with oxygen and moisture levels below 0.01 parts per million. First, TMD monolayers are obtained by micromechanical cleavage of bulk TMD crystal (HQ graphene) onto a 285 nm SiO_2_/Si substrate. hBN crystals were exfoliated onto a poly(dimethylsiloxane) (PDMS) stamp and then used to pick up TMD monolayers. The hBN thickness is typically 20–40 nm. Second, TSC crystals are grown between the sapphire or Si substrates inside a vacuum chamber at room temperature for 24 h. (Detailed structural and ferroelectric characterization data can be found in our previous work^18^ and Supplementary Fig. S1.) Third, the pre-prepared hBN-TMD stack is brought into contact with the TSC crystal carefully. During the transfer process, both mechanical force and heat are minimized to achieve a high-quality interface. In addition, the quality of the cleaved surface of the TSC crystal is double-checked by PFM before transfer (details of PFM and atomic AFM studies are provided in SI). For gate devices, few-layer graphene flakes are exfoliated on a PDMS stamp for dry transfer and used as top and contact gate electrodes. After fabrication, heterostructures are covered by hexagonal boron nitride (hBN) crystals to prevent environmental degradation during the sample loading process.

### Optical measurements

Photoluminescence (PL) measurements were conducted using two cryostats: the attoDRY2100 and the attoDRY800, operating at base temperatures of 1.6 K and 4.2 K, respectively. The attoDRY2100 cryostat was equipped with a superconducting coil capable of generating magnetic fields up to 9 T. Sample cooling in the attoDRY2100 was achieved using helium exchange gas, while the attoDRY800 used thermal contact with a cold finger for cooling. The sample was mounted on a chip carrier positioned on x/y piezo-positioners for precise alignment. An in-situ objective with a numerical aperture of 0.82 was fixed to a *z* piezo-positioner, enabling accurate focalization of laser light onto the device surface. The optical signal was collected and dispersed using a 0.75 m spectrometer equipped with gratings of 150, 600, and 1800 g/mm. Detection was performed using a liquid nitrogen-cooled charge-coupled device (CCD) camera. Magnetic states were probed with the magnetic field applied both in-plane and out-of-plane relative to the c-axis of WSe_2_. A fiber-based probe was used for these measurements. Polarization-resolved experiments (detection) were conducted with the help of polarizers and quarter-wave plates. The excitation energy was tuned close to the bright exciton emission resonance using a supercontinuum source, with an excitation power of 10 μW focused on the sample. Gated structures were characterized using a Keithley 2400 source meter.

Differential reflectance (DR) measurements were performed using a laser confocal system with an ST-500 Janis cryostat at 10 K. A tungsten-halogen lamp was used as the white light source. The light was focused through a lens and directed into an objective lens (Olympus SLMPLN 100×, NA 0.6), producing a beam with a size of approximately 1 μm. The reflected signal was collected and analyzed using the Andor Solis spectrometer equipped with an iDus Si CCD.

The second harmonic generation (SHG) measurement was carried out at room temperature in a home-built multiphoton microscope, pumping with an ultrafast laser (Toptica FemtoFiber Pro NIR, 1550 nm, 90 fs, 80 MHz) through an objective lens (Olympus LCPLN100XIR). The signal was collected in the back-scattering geometry with the same lens. For polarization-resolved SHG, co-polarized excitation and detection beams were used in a normal incidence configuration. A rotating achromatic half-waveplate (Thorlabs SAHWP05M-700) was used to control both the pump laser and signal linear polarization angles, before being analyzed with a fixed polarizer and detected by a spectrometer (Andor Shamrock 500i and iKon L BV).

Raman measurements were conducted at room temperature using a WITec alpha300R confocal Raman microscope system equipped with 532 nm laser excitation. A 50× objective lens was used to focus the laser spot on the sample surface to a diameter of around 1 μm. A spectral grating with 1800 lines/mm was used for collecting Raman spectra.

## Supplementary information

Supplementary Information

Transparent Peer Review file

## Data Availability

All data supporting the findings of this study are included in the main text and the supplementary information file, or from the corresponding author upon request.

## References

[1] Wang, G. et al. Colloquium: excitons in atomically thin transition metal dichalcogenides. *Rev. Mod. Phys.* **90**, 021001 (2018).

[2] Wang, G. et al. In-plane propagation of light in transition metal dichalcogenide monolayers: optical selection rules. *Phys. Rev. Lett.* **119**, 047401 (2017). 29341750 10.1103/PhysRevLett.119.047401

[3] Molas, M. R. et al. Probing and manipulating valley coherence of dark excitons in monolayer WSe_2_. *Phys. Rev. Lett.* **123**, 096803 (2019). 31524465 10.1103/PhysRevLett.123.096803

[4] Robert, C. et al. Measurement of the spin-forbidden dark excitons in MoS_2_ and MoSe_2_ monolayers. *Nat. Commun.* **11**, 4037 (2020). 32788704 10.1038/s41467-020-17608-4 PMC7423942

[5] Robert, C. et al. Fine structure and lifetime of dark excitons in transition metal dichalcogenide monolayers. *Phys. Rev. B* **96**, 155423 (2017).

[6] Slobodeniuk, A. O. & Basko, D. M. Spin–flip processes and radiative decay of dark intravalley excitons in transition metal dichalcogenide monolayers. *2D Mater.* **3**, 035009 (2016).

[7] Zhang, X.-X. et al. Magnetic brightening and control of dark excitons in monolayer WSe_2_. *Nat. Nanotechnol.* **12**, 883–888 (2017). 28650442 10.1038/nnano.2017.105

[8] Molas, M. R. et al. Brightening of dark excitons in monolayers of semiconducting transition metal dichalcogenides. *2D Mater.* **4**, 021003 (2017).

[9] Kipczak et al. Interplay between charge transfer and magnetic proximity effects in WSe_2_/CrCl_3_ heterostructures. *Nanoscale Horiz.* **10**, 2465–2474 (2025). 40747559 10.1039/d5nh00198f

[10] Zhou, Y. et al. Probing dark excitons in atomically thin semiconductors via near-field coupling to surface plasmon polaritons. *Nat. Nanotechnol.* **12**, 856–860 (2017). 28650440 10.1038/nnano.2017.106

[11] Quan, J. et al. On-site enhancement and control of spin-forbidden dark excitons in a plasmonic heterostructure. *Nat. Photonics* **20**, 49–54 (2025).

[12] Sun, J. et al. Routing the Plasmon-Brightened Emission of Dark Excitons in Monolayer WSe_2_ at Room Temperature. *Nano Lett.* **25**, 16757–16763 (2025). 41229321 10.1021/acs.nanolett.5c04709

[13] Jin, S. et al. Plasmonic tuning of dark-exciton radiation dynamics and far-field emission directionality in monolayer WSe2. *Sci. Adv.* **12**, eaea5781 (2026). 41544158 10.1126/sciadv.aea5781 PMC12810645

[14] Park, K.-D., Jiang, T., Clark, G., Xu, X. & Raschke, M. B. Radiative control of dark excitons at room temperature by nano-optical antenna-tip Purcell effect. *Nat. Nanotechnol.* **13**, 59–64 (2018). 29158602 10.1038/s41565-017-0003-0

[15] Kormányos, A., Zólyomi, V., Drummond, N. D. & Burkard, G. Spin-orbit coupling, quantum dots, and qubits in monolayer transition metal dichalcogenides. *Phys. Rev. X* **4**, 011034 (2014).

[16] Dery, H. & Song, Y. Polarization analysis of excitons in monolayer and bilayer transition-metal dichalcogenides. *Phys. Rev. B* **92**, 125431 (2015).

[17] Yang, M. et al. Exciton valley depolarization in monolayer transition-metal dichalcogenides. *Phys. Rev. B* **101**, 115307 (2020).

[18] Wu, Z. et al. Intercalation-driven ferroelectric-to-ferroelastic conversion in a layered hybrid perovskite crystal. *Nat. Commun.* **13**, 3104 (2022). 35662239 10.1038/s41467-022-30822-6 PMC9166815

[19] Hsu, W.-T. et al. Second harmonic generation from artificially stacked transition metal dichalcogenide twisted bilayers. *ACS Nano* **8**, 2951–2958 (2014). 24568359 10.1021/nn500228r

[20] Qian, Q. et al. Chirality-dependent second harmonic generation of mos2 nanoscroll with enhanced efficiency. *ACS Nano* **14**, 13333–13342 (2020). 32926617 10.1021/acsnano.0c05189

[21] Wang, X. et al. Pressure-induced iso-structural phase transition and metallization in WSe_2_. *Sci. Rep.* **7**, 46694 (2017). 28470169 10.1038/srep46694 PMC5415762

[22] Bhatt, S. V., Deshpande, M. P., Sathe, V., Rao, R. & Chaki, S. H. Raman spectroscopic investigations on transition-metal dichalcogenides MX_2_ (M = Mo, W; X = S, Se) at high pressures and low temperature. *J. Raman Spectrosc.* **45**, 971–979 (2014).

[23] Li, H. et al. Mechanical exfoliation and characterization of single- and few-layer nanosheets of WSe_2_, TaS_2_, and TaSe_2_. *Small* **9**, 1974–1981 (2013). 23281258 10.1002/smll.201202919

[24] Zeng, H. et al. Optical signature of symmetry variations and spin-valley coupling in atomically thin tungsten dichalcogenides. *Sci. Rep.* **3**, 1608 (2013). 23575911 10.1038/srep01608 PMC3622914

[25] Koperski, M. et al. Orbital, spin and valley contributions to Zeeman splitting of excitonic resonances in MoSe_2_, WSe_2_ and WS_2_ Monolayers. *2D Mater.* **6**, 015001 (2019).

[26] Arora, A. et al. Zeeman spectroscopy of excitons and hybridization of electronic states in few-layer WSe_2_, MoSe_2_ and MoTe_2_. *2D Mater.* **6**, 015010 (2019).

[27] Koperski, M. et al. Optical properties of atomically thin transition metal dichalcogenides: Observations and puzzles. *Nanophotonics* **6**, 1289–1308 (2017).

[28] Howarth, J. et al. Electroluminescent vertical tunneling junctions based on WSe_2_ monolayer quantum emitter arrays: exploring tunability with electric and magnetic fields. *Proc. Natl. Acad. Sci.* **121**, e2401757121 (2024). 38820004 10.1073/pnas.2401757121 PMC11161753

[29] Koperski, M. et al. Single photon emitters in exfoliated WSe_2_ structures. *Nat. Nanotechnol.* **10**, 503–506 (2015). 25938573 10.1038/nnano.2015.67

[30] Baranowski, M. et al. Dark excitons and the elusive valley polarization in transition metal dichalcogenides. *2D Mater.* **4**, 025016 (2017).

[31] Echeverry, J. P., Urbaszek, B., Amand, T., Marie, X. & Gerber, I. C. Splitting between bright and dark excitons in transition metal dichalcogenide monolayers. *Phys. Rev. B* **93**, 121107 (2016).

[32] Arora, A. et al. Excitonic resonances in thin films of WSe_2_: from monolayer to bulk material. *Nanoscale* **7**, 10421–10429 (2015). 25998778 10.1039/c5nr01536g

[33] Arora, A., Nogajewski, K., Molas, M., Koperski, M. & Potemski, M. Exciton band structure in layered MoSe_2_: from a monolayer to the bulk limit. *Nanoscale* **7**, 20769–20775 (2015). 26603094 10.1039/c5nr06782k

[34] Kane, C. L. & Mele, E. J. Quantum spin hall effect in graphene. *Phys. Rev. Lett.* **95**, 226801 (2005). 16384250 10.1103/PhysRevLett.95.226801

[35] Kane, C. L. & Mele, E. J. Z2 topological order and the quantum spin hall effect. *Phys. Rev. Lett.* **95**, 146802 (2005). 16241681 10.1103/PhysRevLett.95.146802

[36] Mecklenburg, M. & Regan, B. C. Spin and the honeycomb lattice: lessons from graphene. *Phys. Rev. Lett.* **106**, 116803 (2011). 21469887 10.1103/PhysRevLett.106.116803

[37] Kormányos, A. et al. Corrigendum: k.p theory for two-dimensional transition metal dichalcogenide semiconductors (2015 2D Mater. 2 022001). *2D Mater.* **2**, 049501 (2015).

[38] Liu, G.-B., Shan, W.-Y., Yao, Y., Yao, W. & Xiao, D. Three-band tight-binding model for monolayers of group-VIB transition metal dichalcogenides. *Phys. Rev. B* **88**, 085433 (2013).

[39] Xiao, D., Liu, G.-B., Feng, W., Xu, X. & Yao, W. Coupled spin and valley physics in monolayers of MoS_2_ and other group-VI dichalcogenides. *Phys. Rev. Lett.* **108**, 196802 (2012). 23003071 10.1103/PhysRevLett.108.196802

[40] Zinkiewicz, M. et al. Neutral and charged dark excitons in monolayer WS_2_. *Nanoscale* **12**, 18153–18159 (2020). 32853305 10.1039/d0nr04243a

[41] Jones, A. M. et al. Optical generation of excitonic valley coherence in monolayer WSe_2_. *Nat. Nanotechnol.* **8**, 634–638 (2013). 23934096 10.1038/nnano.2013.151

