# Supplementary material for: Ferroelectric brightening of spin‑forbidden dark excitons in a WSe2/hybrid-perovskite heterostructure
Source: Nat Commun. 2026 May 9;17:6285. doi: 10.1038/s41467-026-72143-y (PMC13376157; doi:10.1038/s41467-026-72143-y)
Supplement: Supplementary file 1 — Supplementary Information [file 41467_2026_72143_MOESM1_ESM.pdf]

Ferroelectric brightening of spin-forbidden dark exciton in a WSe_2/hybrid-perovskite heterostructure
Supplementary Information

Xinyun Wang[1,2†], Magdalena Grzeszczyk[3†], Maxim Trushin[3,4,5†], Ivan Verzhbitskiy[6,7], Dmitrii Litvinov[3], Yi Wei Ho[2,3], Yuan Chen[1], Zhenyue Wu[1], Mykola Telychko[1], Chuanqi Zhang[1], Andres Granados del Aguila[3], Kuan Eng Johnson Goh[2,6,7,8], Xinwei Li[2], Goki Eda[1,2,4], Shaffique Adam[2,4], Maciej Koperski[3]*, and Kian Ping Loh[1]*

[1]Department of Chemistry, National University of Singapore, Singapore, Singapore.

[2]Department of Physics, National University of Singapore, Singapore, Singapore.

[3]Institute for Functional Intelligent Materials, National University of Singapore, Singapore, Singapore.

[4]Centre for Advanced 2D Materials and Graphene Research Centre, National University of Singapore, Singapore, Singapore.

[5]Department of Materials Science Engineering, National University of Singapore, Singapore, Singapore.

[6]Quantum Innovation Center, Agency for Science Technology and Research, Singapore, Singapore.

[7]Institute of Materials Research and Engineering, Agency for Science Technology and Research, Singapore, Singapore.

[8]Division of Physics and Applied Physics, School of Physical and Mathematical Sciences, Nanyang Technological University, Singapore, Singapore.

[†]Equal contribution to this work.

Table of Content

Section 1: Polarization-dependent SHG pattern fitting.

Section 2: Details of theoretical studies.

1. Tight-binding model of the proximity-induced spin flip.
2. Twist-angle-dependent SOC and dipole transition strength.
3. Symmetry analysis of polarization properties of the dark-grey exciton doublet.

Section 3: Additional results of experimental data.

1. Fig. S1 | Co-polarized SHG polar plot of zigzag- and armchair-stacked heterostructures.
2. Fig. S2 | Directional control of SOC on a honeycomb lattice.
3. Fig. S3 | Twist-angle-dependent SOC.
4. Fig. S4 | Sketch of the electronic band structures and exciton fine structures for monolayer WSe_2.
5. Fig. S5 | Twist-angle-dependent phase difference of the dark-grey exciton doublet.
6. Fig. S6 | Lattice structure characterization and ferroelectric polarization switching in perovskite TSC.
7. Fig. S7 | Symmetry breaking induced by the other ferroelectric material.
8. Fig. S8 | Differential-reflectance spectra.
9. Fig. S9 | Out-of-plane magnetic-field-dependent PL spectra in a heterostructure device.
10. Fig. S10 | Comparison of PL spectra from heterostructures with other TMDs.
11. Fig. S11 | Gate dependence of the emerged dark excitons in a heterostructure device.
12. Fig. S12 | Power dependence of the dark exciton from a heterostructure.
13. Fig. S13 | In-plane magnetic field dependence of PL spectra from a defect site in a WSe_2 monolayer.
14. Fig. S14 | Time-trace PL emission of dark excitons.
15. Fig. S15 | In-plane spin texture of topmost valance band.
16. Fig. S16 | Reduced magneto-brightening curvature α with increasing twist angle.

Section 1: Polarization-dependent SHG pattern fitting.

We first consider the fitting for individual crystals. In the electric dipole approximation, the second harmonic field of each single crystal can be calculated from the second-order nonlinear tensors under the constraints of the corresponding crystallographic symmetry[1]. The TSC perovskite has the C_{1h} (C_s) lattice symmetry[2] and its second-order nonlinear tensor can be expressed as:

$$\begin{pmatrix} \begin{pmatrix}\chi_{xxx}\\0\\\chi_{xxz}\end{pmatrix} & \begin{pmatrix}0\\\chi_{xyy}\\0\end{pmatrix} & \begin{pmatrix}\chi_{xzx}\\0\\\chi_{xzz}\end{pmatrix} \\ \begin{pmatrix}0\\\chi_{yxy}\\0\end{pmatrix} & \begin{pmatrix}\chi_{yyx}\\0\\\chi_{yyz}\end{pmatrix} & \begin{pmatrix}0\\\chi_{yzy}\\0\end{pmatrix} \\ \begin{pmatrix}\chi_{zxx}\\0\\\chi_{zxz}\end{pmatrix} & \begin{pmatrix}0\\\chi_{zyy}\\0\end{pmatrix} & \begin{pmatrix}\chi_{zzx}\\0\\\chi_{zzz}\end{pmatrix} \end{pmatrix}$$

In a normal incidence configuration, the SHG signal with a parallel polarization geometry to the incident laser beam is given by:

$$I_{(2\omega,\theta)}^{TSC} \propto \left|(\chi_{xxx}\cos^3\theta + (\chi_{xyy} + \chi_{yxy} + \chi_{yyx})\cos\theta\sin^2\theta)^2\right|$$

where θ is the angle between the polarization axis of incoming/outgoing light and the TSC perovskite c axis.

The WSe_2 monolayer has the D_{3h} lattice symmetry[3,4] and its second-order nonlinear tensor can be expressed as:

$$\begin{pmatrix} \begin{pmatrix}0\\-\chi_{yyy}\\0\end{pmatrix} & \begin{pmatrix}-\chi_{yyy}\\0\\0\end{pmatrix} & \begin{pmatrix}0\\0\\0\end{pmatrix} \\ \begin{pmatrix}-\chi_{yyy}\\0\\0\end{pmatrix} & \begin{pmatrix}0\\\chi_{yyy}\\0\end{pmatrix} & \begin{pmatrix}0\\0\\0\end{pmatrix} \\ \begin{pmatrix}0\\0\\0\end{pmatrix} & \begin{pmatrix}0\\0\\0\end{pmatrix} & \begin{pmatrix}0\\0\\0\end{pmatrix} \end{pmatrix}$$

where $\chi_{yyy} = -\chi_{yxx} = -\chi_{xxy} = -\chi_{xyx}$.

Similarly, the second harmonic intensity can be derived as:

$$I_{(2\omega,\theta)}^{WSe_2} \propto \left|(\chi_{yyy} \sin^3 \theta)^2\right|$$

Next, we consider the fitting for heterostructure. Upon the previous SHG fitting for individual single crystal, the twist angle of the heterostructure has been determined. If no additional symmetry is breaking at the interface (C_{1h} and D_{3h} symmetries are preserved), the second harmonic intensity for the heterostructure can be achieved using the linear combination of the two independent second harmonic fields, which can be expressed as:

$$I_{(2\omega,\theta)}^{HS} \propto \left|(\chi_{tscxxx} \cos^3(\theta + \varphi) + (\chi_{tscxyy} + \chi_{tscyxy} + \chi_{tscyyx}) \cos(\theta + \varphi) \sin^2(\theta + \varphi) + \chi_{WSe_2yyy} \sin^3(\theta))^2\right|$$

where φ is the angle between the WSe_2 zigzag axis and the TSC ferroelectric polarization axis. Since the twist angle φ can be determined by the previous SHG fitting, it is no longer a variable in the fitting process for heterostructure. Notably, we found that this linear combination function cannot fit the SHG polar patterns of different twisting-angle heterostructures. Moreover, the SHG intensity from heterostructure is at least twofold higher than that from each individual component. The observations above validate the additional symmetry breaking in the heterostructures.

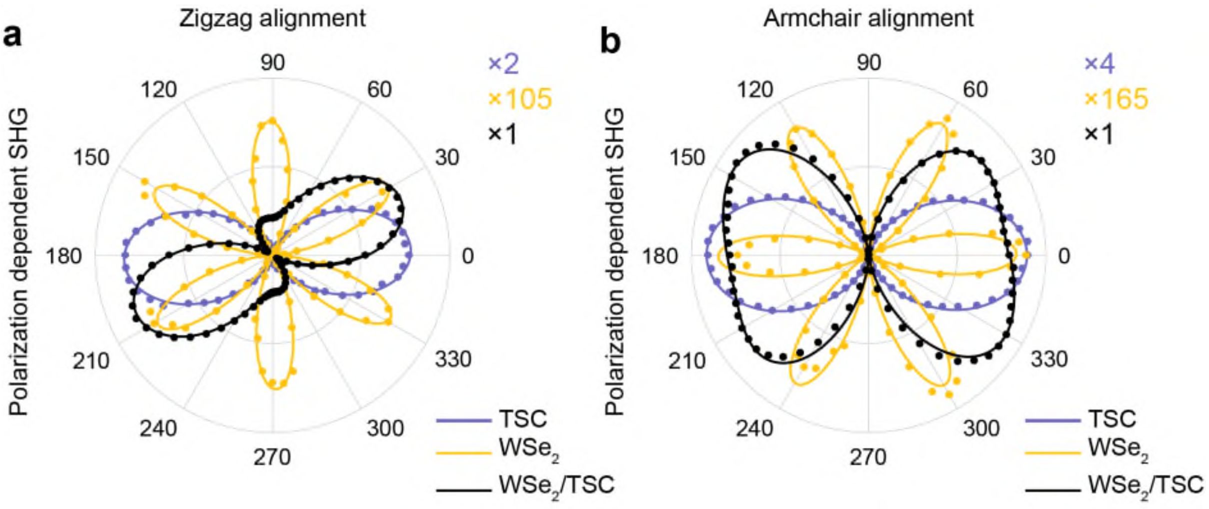

Fig. S1 | Co-polarized SHG polar plot of zigzag- and armchair-stacked heterostructures. a, b, Corresponding polarization-dependent SHG patterns from the two heterostructures and individual layers, confirming the zigzag and armchair alignments. The data for individual layers is fitted using their pristine symmetry, which is D_{3h} for WSe_2 and C_{1h} (C_s) for TSC perovskite. The data for heterostructure is fitted using the C_1 symmetry for WSe_2 and C_{1h} symmetry for TSC perovskite.

In order to reveal the new symmetry of the heterostructures, we tested all the subgroups under D_{3h} symmetry and found that only C_1 symmetry can fulfill the SHG patterns with different alignment, as shown in Fig. T1. The second harmonic intensity under C_1 (WSe_2) and C_{1h} (TSC) symmetry can be derived as below:

$$
\begin{aligned}
I^{HS}_{(2\omega,\theta)} \propto \Big| (\chi_{tscxxx}\cos^3(\theta+\varphi) + \left(\chi_{tscxyy}+\chi_{tscyxy}+\chi_{tscyyx}\right)\cos(\theta+\varphi)\sin^2(\theta+\varphi) \\
+ \chi_{WSe_2xxx}\cos^3\theta + \left(\chi_{WSe_2xxy}+\chi_{WSe_2xyx}+\chi_{WSe_2yxx}\right)\cos^2\theta\sin\theta \\
+ \left(\chi_{WSe_2xyy}+\chi_{WSe_2yxy}+\chi_{WSe_2yyx}\right)\cos\theta\sin^2\theta + \chi_{WSe_2yyy}\sin^3\theta)^2 \Big|
\end{aligned}
$$

Considering the emission wavelengths of SHG may involve the resonant electronic transitions in WSe_2, the eight independent tensor elements are replaced with complex numbers in the fitting. Here we note that, although the horizontal mirror reflection (σ_h) symmetry breaking cannot be identified in a normal incident SHG measurement, the heterostructure configuration of a single-sided substrate already destroys σ_h (top and bottom are not equivalent). Therefore, with combination of three-fold rotational and horizontal mirror symmetry breaking, no symmetry is present in the heterostructure (C_1).

Section 2: Details of theoretical studies.

1. Tight-binding model of the proximity-induced spin flip.

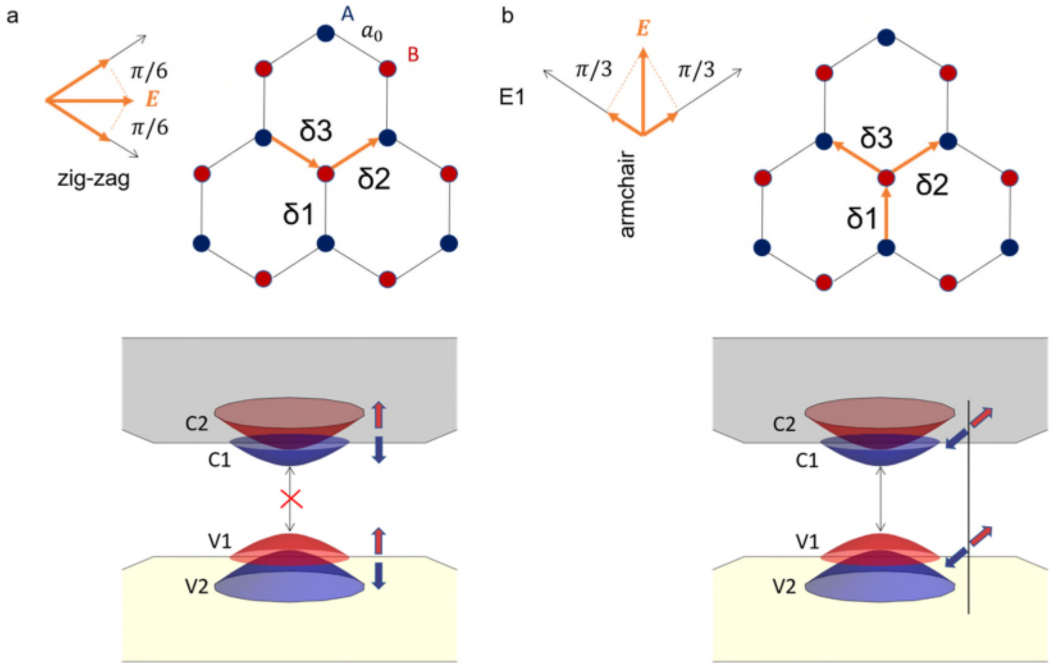

Fig. S2 | Directional control of SOC on a honeycomb lattice. a, For a zigzag-oriented field, the non-vanishing electric field projections are the same for A→B and B→A hopping paths. The SOC is equivalent for the opposite hopping paths, keeping conduction- and valence-band spins antiparallel and the optical transition dark. b, The macroscopic electric field is along one of the armchair directions with the projections different for A→B and B→A hopping paths. This makes the SOC depends on the electron hopping direction, reorienting the spins and enabling the previously spin-forbidden optical transitions.

In this section, we provide an intuitive theoretical model based on tight-binding model to capture the dominant features of our results. In a pristine WSe_2 monolayer (point group D_{3h} with horizontal mirror symmetry σ_h), the band-edge spins are z-polarized (Ising-like) and any in-plane component is forbidden by σ_h symmetry. At small perturbation limit, where σ_h is weakly broken by asymmetric dielectric environments (two sides are different) or an external out-of-plane electric field in a gated device, the spin-flip term in the off-diagonal matrix is allowed and the in-plane spin component can be present. However, the resulting Rashba-type spin flip remains negligible because the Rashba parameter is k-dependent and vanishes at the

band extrema[5,6]. Another way to induce spin flipping is to modify the intersublattice spin-dependent hopping through Kane-Mele (KM) spin-orbit coupling (SOC). However, under the threefold rotational symmetry C_3, the nearest-neighbour hoppings have identical amplitudes along all three bond directions, and the spin flips acquired along different paths cancel out, leaving the conduction- and valence-band spins antiparallel and the ground-state exciton dark.

To generate spin flipping and enable the dark exciton transition, two symmetries must be violated:1. broken σ_h to allow in-plane spin polarization; 2. reduced C_3 to allow inequivalent SOC strengths for different hopping pathways (sublattice-asymmetric SOC). Both requirements are naturally achieved in our ferroelectric heterostructure, where σ_h is broken by the interface itself and C_3 is further reduced by the in-plane ferroelectric field. To formalize the problem, we employ a low-energy four-band Hamiltonian for electrons on a honeycomb lattice with spin-orbit interactions reads:

$$H_0 = \sum_{\mathbf{k}} \left(a^{\dagger}_{\mathbf{k}\uparrow} b^{\dagger}_{\mathbf{k}\uparrow} a^{\dagger}_{\mathbf{k}\downarrow} b^{\dagger}_{\mathbf{k}\downarrow}\right) \begin{pmatrix} \frac{\Delta_0}{2} & \hbar v k e^{-i\varphi_{\mathbf{k}}} & \frac{\Delta_{sf}}{2} & \hbar v_{AB} k e^{-i\varphi_{\mathbf{k}}} \\ \hbar v k e^{i\varphi_{\mathbf{k}}} & -\frac{\Delta_0}{2} & \hbar v_{BA} k e^{i\varphi_{\mathbf{k}}} & \frac{\Delta_{sf}}{2} \\ \frac{\Delta_{sf}}{2} & \hbar v_{BA} k e^{-i\varphi_{\mathbf{k}}} & \frac{\Delta_0}{2} & \hbar v k e^{-i\varphi_{\mathbf{k}}} \\ \hbar v_{AB} k e^{i\varphi_{\mathbf{k}}} & \frac{\Delta_{sf}}{2} & \hbar v k e^{i\varphi_{\mathbf{k}}} & -\frac{\Delta_0}{2} \end{pmatrix} \begin{pmatrix} a_{\mathbf{k}\uparrow} \\ b_{\mathbf{k}\uparrow} \\ a_{\mathbf{k}\downarrow} \\ b_{\mathbf{k}\downarrow} \end{pmatrix}$$

Here $a^{\dagger}_{\mathbf{k}\boldsymbol{\sigma}}$ ($b^{\dagger}_{\mathbf{k}\boldsymbol{\sigma}}$) is an electron creation operator with the wave vector $\mathbf{k}$ ($|\mathbf{k}| = k$) and spin index $\boldsymbol{\sigma} = \{\uparrow, \downarrow\}$ on sublattice A(B), $v = 3|t|a_0/2$ with t and a_0 being respectively the nearest-neighbor hopping parameter and distance, $\varphi_{\mathbf{k}}$ is the direction of $\mathbf{k}$, and $\hbar$ is the reduced Planck constant. The hopping terms have been expanded near one of the corners of the first Brillouin zone (K-valley), and the Hamiltonian for the opposite valley is just a time-reversal of H_0. The bandgap Δ_0 opens because of the on-site energy difference between sublattices A and B (i.e. the sublattices are occupied by different atoms, W and a pair of Se in our case). Spin flip process is described phenomenologically by the off-diagonal terms between the spin-up and spin-down blocks. The on-site spin-flip energy in presence of the external electric field is described by Δ_{sf}.

The inter-site spin-flip terms are described by parameters v_{AB} and v_{BA}, which can be quantified as $v_{AB} \sim \hbar e E_{AB}/m^* \Delta_0$ and $v_{BA} \sim \hbar e E_{BA}/m^* \Delta_0$, where E_{AB} and E_{BA} are the potential gradients along the A→B and B→A links on the honeycomb lattice, as shown in Fig. T2. E_{AB} and E_{BA} can be regarded the macroscopic electric field projections on the respective links between A and B sites. The effective electron mass is determined by the parameters Δ_0 and v, estimated as $m^* = 2\Delta_0/v^2$ and is about the same for all bands ($0.3 - 0.5m_0$)[7]. The momentum dependence of all the hopping terms is the same ($\propto k e^{\pm i\varphi_k}$) because the hopping occurs along the same links regardless spin-flip. Diagonalizing H_0 we obtain two spin-splitting conduction bands having bottoms at the energy levels $\frac{\Delta_0+\Delta_{sf}}{2}$(C2) and $\frac{\Delta_0-\Delta_{sf}}{2}$ (C1), as well as two spin-splitting valence bands capped at $-\frac{\Delta_0-\Delta_{sf}}{2}$ (V1) and $-\frac{\Delta_0+\Delta_{sf}}{2}$(V2), as depicted in Fig. T2. The zero-energy level is placed in the middle of the bandgap.

To illustrate the effect of asymmetrical sublattice hopping strength on spin orientation, we consider two cases in which the in-plane **E** field is aligned either along the zigzag or along the armchair axis of the WSe_2 lattice. For zigzag alignment, its projections onto the three nearest-neighbour hopping links are:

$$\mathbf{E} \cdot \boldsymbol{\delta}_1 = 0, \qquad \mathbf{E} \cdot \boldsymbol{\delta}_2 = +\frac{\sqrt{3}}{2} E_0 d, \qquad \mathbf{E} \cdot \boldsymbol{\delta}_3 = -\frac{\sqrt{3}}{2} E_0 d$$

where the nearest-neighbour vectors (length d) is expressed as: $\boldsymbol{\delta}_1 = (0, -d)$, $\boldsymbol{\delta}_2 = (\frac{\sqrt{3}}{2} d, \frac{1}{2} d)$, $\boldsymbol{\delta}_3 = (-\frac{\sqrt{3}}{2} d, \frac{1}{2} d)$, as illustrated in Fig. T2. In this configuration, the set of projections on the hopping links is $\{0, +p, -p\}$ with $p = \frac{\sqrt{3}}{2} E_0 d$. The hopping links have identical strength for v_{AB} and v_{BA}. Any asymmetrical sublattice hopping that depends on summing these projections over the three bonds therefore vanishes. Notably, even with a nonzero onsite spin-flip energy, spin orientation is absent and the lowest-energy transition remains spin-forbidden.

In contrast, when E is projected along the armchair axis, the field projections become:

$$\mathbf{E} \cdot \boldsymbol{\delta}_1 = +E_0 d, \qquad \mathbf{E} \cdot \boldsymbol{\delta}_2 = +\frac{1}{2} E_0 d, \qquad \mathbf{E} \cdot \boldsymbol{\delta}_3 = -\frac{1}{2} E_0 d$$

the set of projections on the hopping links is $\{+p, +\frac{1}{2}p, -\frac{1}{2}p\}$ with $p = E_0 d$. The hopping asymmetry survives, hence, altering the relative spin orientation of hopping electrons and generating in-plane spin textures, as shown in Fig. 4. Therefore, the band-edge spins are no

longer strictly antiparallel, allowing a lifting of the spin selection rule and thereby enabling optical transitions.

2. Twist-angle-dependent SOC and dipole transition strength.

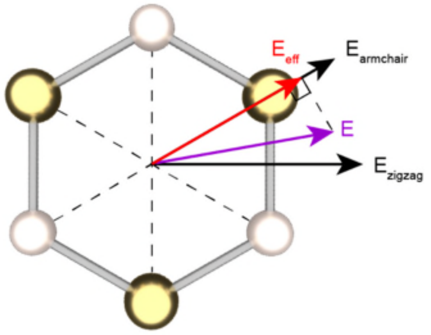

Fig. S3 | Twist-angle-dependent SOC. Schematic diagram and plot of the twist-angle-dependent effective electric field E_{eff}, where the E field is the macroscopic electric field imprinted from the underlying ferroelectric substrate.

In this section, we quantify the twist-angle-dependent SOC and the corresponding dipole transition strength. Following the discussion above, the magnitude of the SOC field depends on the electron hopping direction, which is controlled by the imprinted ferroelectric field. To describe this relationship quantitatively, we first define the effective electric field E_{eff} as the net field component projected along the armchair hopping links of the honeycomb lattice. This field determines the degree of sublattice-asymmetric spin-flip hopping and thus directly controls the strength of the induced in-plane SOC:

$$E_{eff} = E\cos(\theta - \frac{\pi}{6}) - \frac{1}{2}E\cos\left(\theta + \frac{\pi}{6}\right) - \frac{1}{2}E\sin\theta \approx E\sin\theta, \qquad \theta \in (-\frac{\pi}{6}, \frac{\pi}{6})$$

here E is the macroscopic electric field imprinted from the underlying ferroelectric substrate and θ is the twist angle of the E field from the zigzag axis (Fig. T3). The difference of the twist-angle dependent spin-orbit parameter v_{diff} can thus be expressed in terms of effective electric field:

$$v_{diff} = |v_{BA} - v_{AB}| = \frac{\hbar e E_{eff}}{m^* \Delta_0} = \frac{\hbar e E}{m^* \Delta_0} \sin\theta$$

where $\hbar$ is the reduced Planck constant, e is the elementary charge, $m^*$is the effective electron mass (roughly taken as $0.4m_0$), and Δ_0 is the band gap.

To simplify the calculation, we let $v_{BA} = -v_{AB} = \frac{1}{2} v_{diff} = \frac{\hbar e E}{2m^* \Delta_0} \sin\theta$. By substituting the twist-angle-dependent effective field into H_0, we derive the velocity operator and the light-matter interaction Hamiltonian then reads:

$$H_1 = \frac{e}{2c} |A| \sin\theta \sum_{\mathbf{k}} \left(a^\dagger_{\mathbf{k}\uparrow} b^\dagger_{\mathbf{k}\uparrow} a^\dagger_{\mathbf{k}\downarrow} b^\dagger_{\mathbf{k}\downarrow} \right) \begin{pmatrix} 0 & ve^{-i\varphi_{\mathbf{A}}} & 0 & v_{AB} e^{-i\varphi_{\mathbf{A}}} \\ ve^{i\varphi_{\mathbf{A}}} & 0 & v_{BA} e^{i\varphi_{\mathbf{A}}} & 0 \\ 0 & v_{BA} e^{-i\varphi_{\mathbf{A}}} & 0 & ve^{-i\varphi_{\mathbf{A}}} \\ v_{AB} e^{i\varphi_{\mathbf{A}}} & 0 & ve^{i\varphi_{\mathbf{A}}} & 0 \end{pmatrix} \begin{pmatrix} a_{\mathbf{k}\uparrow} \\ b_{\mathbf{k}\uparrow} \\ a_{\mathbf{k}\downarrow} \\ b_{\mathbf{k}\downarrow} \end{pmatrix}$$

Where the $\mathbf{A}(\omega, \mathbf{q})$ is the vector potential to describe the electromagnetic wave emitted due to electron-hole recombination and included in H_0 by a substitution $\mathbf{k} \rightarrow \mathbf{k} - \frac{e}{\hbar c} \mathbf{A}(\omega, \mathbf{q})$, $\varphi_{\mathbf{A}}$ is the light polarization angle, e is the elementary charge, c is the speed of light, ω and q are the electromagnetic wave frequency and wave vector, respectively.

The interband optical transitions can be evaluated using Fermi's golden rule, based on the eigenfunctions of the unperturbed Hamiltonian H_0. The lowest-energy transition probability is proportional to the square of the dipole matrix element:

$$|M(k)|^2 = |\langle CB1|H_1|VB1\rangle|^2$$

where CB1and VB1 are the eigenfunctions of the lowest conduction band and highest valence band of the unperturbed Hamiltonian H_0. Consequently, the oscillator strength follows:

$$|M(k)|^2 \propto |\sin\theta|^2$$

Obviously, the optical transition is completely suppressed when the ferroelectric field is aligned along the zigzag axis ($\theta = 0°$) and reaches a maximum when the field is aligned with the armchair direction ($\theta = 30°$), as shown in Fig. 4.

3. Symmetry analysis of polarization properties of the dark-grey exciton doublet.

In this section, we consider the polarization properties of the brightened dark-grey exciton doublet from the heterostructures. Although we have demonstrated the three-fold rotational symmetry breaking induced by the ferroelectric substrate in the main text, it is still instructive to analyze the wavefunctions of the excitonic states in terms of irreducible representations of the D_{3h} point symmetry group with a perturbation correction at relatively weak-field limit.

The conduction and valence band wavefunctions for pristine WSe_2 monolayer can be established in terms of irreducible representations of D_{3h} point group at K^- and K^+ points: $\mathbf{U}^{\gamma}_{\tau,s}(\mathbf{r}) \equiv e^{-i\mathbf{K}_\tau\cdot\mathbf{r}} u^{\gamma}_{\mathbf{K}_\tau\cdot s}(\mathbf{r})$, where $u^{\gamma}_{\mathbf{K}_\tau\cdot s}(\mathbf{r})$ is the conduction/valence band Bloch wavefunction, γ is the invariant representation of D_{3h} point group, τ is the valley index, s is the spin index and $\mathbf{K}_\tau$ is the valley dependent wave vector. Note that the two valleys are coupled by the time-reversal symmetry, which ensures the relation: $\mathcal{K}\left[\mathbf{U}^{\gamma}_{\tau,s}(\mathbf{r})\right] = (-1)^{\frac{1}{2}-s} \cdot \left(\mathbf{U}^{\gamma}_{-\tau,-s}(\mathbf{r})\right)^*$, where $\mathcal{K}$ is the time-reversal operator in case of odd number of electrons. Fig. T4a shows the obtained band wavefunctions at two valleys in a single-particle picture, labeled with the corresponding irreducible representations and arrows for electron spin orientation.

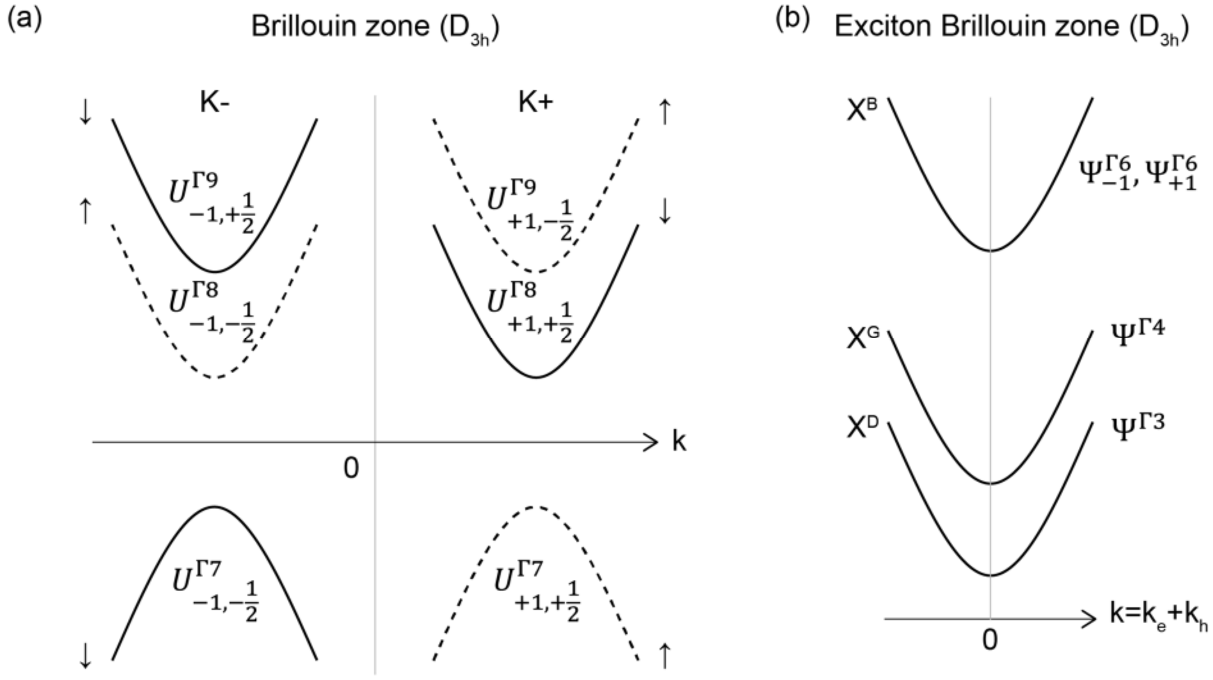

Fig. S4 | Sketch of the electronic band structures and exciton fine structures for monolayer WSe_2. Electrons and exciton wavefunctions are expressed by irreducible representations of D_{3h} point symmetry group.

Next we consider the exciton formation process, which involves the two bottom conduction bands and the topmost valence band. The formed exciton states can be expressed accordingly:

Spin-allowed optical transition: $\Gamma_{X^{\uparrow\uparrow,\downarrow\downarrow}} = \Gamma_7{}^* \times \Gamma_9 = \Gamma_5 + \Gamma_6$,
Spin-forbidden optical transition: $\Gamma_{X^{\uparrow\downarrow,\downarrow\uparrow}} = \Gamma_7{}^* \times \Gamma_8 = \Gamma_3 + \Gamma_4 + \Gamma_6$.

We can obtain the wavefunction for bright exciton with Γ_6 representation, grey exciton with Γ_4 representation, dark exciton with Γ_3 representation, as shown in Fig. T4b. Note that Γ_5 state is momentum-forbidden for $\Gamma_{X^{\uparrow\uparrow,\downarrow\downarrow}}$ transition process and Γ_6 state is also momentum-forbidden for $\Gamma_{X^{\uparrow\downarrow,\downarrow\uparrow}}$ transition process, which is thus omitted here.

Following the coupling tables of D_{3h} group[8] and phase convention[9-12], we can derive the wavefunctions for excitons:

Bright exciton $X^{B}_{\Gamma_6}$: $\Psi^6_{-1} = -\mathcal{K}\left(U^7_{-1,-\frac{1}{2}}\right) \cdot U^9_{-1,\frac{1}{2}} = b^+$,

$$\Psi_1^6 = \mathcal{K}\left(U^7_{1,\frac{1}{2}}\right)\cdot U^9_{1,-\frac{1}{2}} = b^-,$$

Grey exciton $X^G_{\Gamma_4}$: $\Psi^4 = \frac{i}{\sqrt{2}}(\mathcal{K}\left(U^7_{1,\frac{1}{2}}\right)\cdot U^8_{1,\frac{1}{2}} - \mathcal{K}(U^7_{-1,-\frac{1}{2}})\cdot U^8_{-1,-\frac{1}{2}}) = \frac{i}{\sqrt{2}}(d^+ - d^-),$

Dark exciton $X^D_{\Gamma_3}$: $\Psi^3 = \frac{1}{\sqrt{2}}(\mathcal{K}\left(U^7_{1,\frac{1}{2}}\right)\cdot U^8_{1,\frac{1}{2}} + \mathcal{K}(U^7_{-1,-\frac{1}{2}})\cdot U^8_{-1,-\frac{1}{2}}) = -\frac{1}{\sqrt{2}}(d^+ + d^-).$

here $b^{+/-}$ denote the two circularly selective bright excitons (coupling to $\sigma^\pm$ light respectively) and $d^{+/-}$ denote intravalley spin-forbidden states in $K^{+/-}$ valleys. Obviously, $X^G_{\Gamma_4}$ and $X^D_{\Gamma_3}$ have fundamental odd/even valley phase, with a relative phase difference of π.

In the following, we consider how the SOC effect determines the polarization properties of the brightened dark-grey doublet. The SOC mainly has two contributions: 1) bright/dark exciton states mixing which provides the brightening channel (SOC-enabled spin mixing under symmetry breaking); 2) intervalley mixing induced by the sublattice-asymmetric perturbation.

To capture these features, we employ an effective exciton Hamiltonian written in the valley basis (b^+, b^-, d^+, d^-). This basis is convenient because the emitted polarization (Jones vector) is directly set by the bright amplitudes in the $\sigma^\pm$ channels. Importantly, the transformation between the $(\Psi^6_{+1}, \Psi^6_{-1}, \Psi^3, \Psi^4)$ irrep basis and the valley basis is unitary, so inner products of polarization vectors (polarization angles) are basis-independent. The effective exciton Hamiltonian can be presented in the form:

$$H^v_{soc'} = \begin{pmatrix} \Delta & \beta & V & 0 \\ \beta & \Delta & 0 & V \\ V & 0 & 0 & \xi/2 + i\varepsilon \\ 0 & V & \xi/2 - i\varepsilon & 0 \end{pmatrix}$$

where Δ is the on-site spin splitting energy; ξ is the fine splitting between Γ_3 and Γ_4 (short-range exchange interaction); V is the real bright/dark mixing amplitude, proportional to the effective spin-orbit hopping strength; β is the bright valley states (b^+, b^-) mixing parameter and ε is dark valley states (d^+, d^-) mixing parameter. V, β and ε are determined by the

sublattice-dependent orbital mixing and characterize how strongly it modifies the valley-selective optics.

In the limit of $|\Delta| \gg |V|, |\beta|, |\xi|$ and $|\varepsilon|$, where manifold is well separated in energy and the perturbation enters primarily through small admixtures, we can derive the dark-sector eigenstates in the valley basis of (d^+, d^-) by diagonalizing the lower 2×2 block:

$$d'^{+} = \frac{1}{\sqrt{2}}\begin{pmatrix} e^{-i\phi/2} \\ e^{i\phi/2} \end{pmatrix} \text{ and } d'^{-} = \frac{1}{\sqrt{2}}\begin{pmatrix} e^{-i\phi/2} \\ -e^{i\phi/2} \end{pmatrix}$$

Here we define $\cos\phi = \xi/2\sqrt{(\xi/2)^2 + \varepsilon^2}$ and $\sin\phi = \varepsilon/\sqrt{(\xi/2)^2 + \varepsilon^2}$.

The bright-dark mixing parameter V admixes a small bright component into each dark eigenstate. Solving the bright block and we have:

$$b' \cong -H_{BB}^{-1} H_{BD} d', \text{ with } H_{BB} = \begin{pmatrix} \Delta & \beta \\ \beta & \Delta \end{pmatrix} \text{ and } H_{BD} = \begin{pmatrix} V & 0 \\ 0 & V \end{pmatrix}$$

We can obtain the admixed bright states:

$$b'^{+} = \frac{V}{\sqrt{2}(\Delta^2 - \beta^2)}\begin{pmatrix} -\Delta e^{-i\phi/2} + \beta e^{i\phi/2} \\ -\Delta e^{i\phi/2} + \beta e^{-i\phi/2} \end{pmatrix} \text{ and } b'^{-} = \frac{V}{\sqrt{2}(\Delta^2 - \beta^2)}\begin{pmatrix} -\Delta e^{-\frac{i\phi}{2}} - \beta e^{i\phi/2} \\ \Delta e^{i\phi/2} + \beta e^{-i\phi/2} \end{pmatrix}$$

Physically, β describes how strongly the symmetry breaking admixes the two circularly selective bright valleys (mix $\sigma^{\pm}$ channels), while $\xi/2 \pm i\varepsilon$ quantifies an additional dark-sector intervalley coupling channel that rotates the valley-coherent eigenstates away from the pure exchange axis. The parameter V determines the overall brightness (oscillator strength) of the dark/grey doublet.

Since $b'^{\pm}$ correspond to circular polarized emissions $\sigma^{\pm}$, the emitted Jones vectors $P^{\pm}$ in the circular basis are proportional to

$$\mathbf{P}^{\pm} \propto (b'^{+}, b'^{-})$$

Because the transformation between $\Psi^{6}_{\pm1} \leftrightarrow b^{\pm}$ is unitary, we can derive the optical polarization angle φ between grey and dark excitons using the two Jones vectors above:

$$\cos\varphi = \frac{(\mathbf{P}^1)^{\dagger}\cdot\mathbf{P}^2}{|\mathbf{P}^1||\mathbf{P}^2|} \approx 2\frac{\beta}{\Delta}|\sin\phi| = 2\frac{\beta}{\Delta}\frac{\varepsilon}{\sqrt{(\xi/2)^2+\varepsilon^2}}$$

For small deviations from orthogonality, phase shift angle φ can be approximated and established as:

$$\varphi \approx \frac{\pi}{2} - \cos\varphi = \frac{\pi}{2} - 2\frac{\beta}{\Delta}\frac{\varepsilon}{\sqrt{(\xi/2)^2+\varepsilon^2}}$$

Note that the valley mixing parameters β and ε are proportional to the SOC strength. We therefore take β and ε to scale with the effective spin-orbit hopping energy E_{soc}, which is twist-angle dependent and can be expressed:

$$E_{soc}(\theta) = \frac{\hbar\upsilon_{diff}}{a_0} = \frac{\hbar^2 eE}{a_0 m^* \Delta_0}\sin\theta = E_0\sin\theta$$

Therefore, the phase shift angle φ follows a twist-dependent trend through $E_0^{\beta}\sin\theta$ and $E_0^{\varepsilon}\sin\theta$:

$$\varphi = \frac{\pi}{2} - 2\frac{E_0^{\beta}\sin\theta}{\Delta}\frac{E_0^{\varepsilon}\sin\theta}{\sqrt{(\xi/2)^2+(E_0^{\varepsilon}\sin\theta)^2}} \approx \frac{\pi}{2} - 4\frac{E_0^{\beta}E_0^{\varepsilon}}{\Delta\xi}\sin^2\theta$$

here the valley mixing amplitudes are in the form of E_0^{β} and E_0^{ε} (both ∝ E_{soc}).

The in-plane ferroelectric field of TSC crystal is measured by P-V loop and estimated to be about 10^9 V/m at room temperature. Therefore, we could calculate the maximum spin-orbit hopping energy E_0 to be around 400 meV, much larger than the estimated value of 40 meV for Δ and 0.6 meV for ξ. However, the effective exciton-level mixing amplitudes β and ε entering H^{v}_{soc}, are reduced by screening, orbital-projection factors and symmetry-imposed cancellations, and should be understood as phenomenological parameters in the weak-mixing regime $\beta, \varepsilon \ll$

Δ. At a small twist angle limit, we plot the twist-angle-dependent phase shift in Fig. T6. The obtained curve is quite consistent with the reduced phase difference obtained in our polarization dependent PL measurements (Fig. 5) and thus supports our theoretical model.

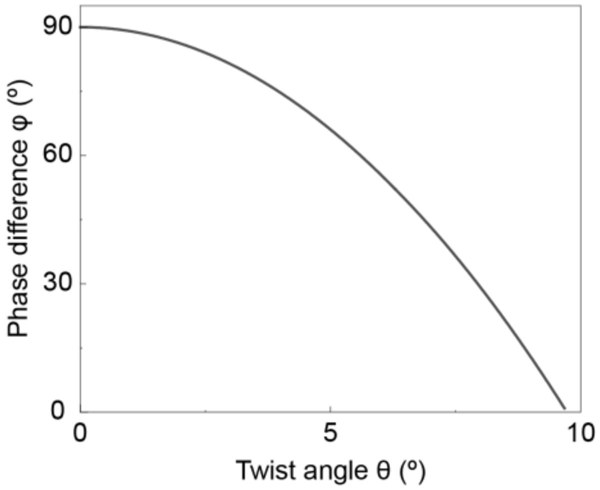

Fig. S5 | Twist-angle-dependent phase difference of the dark-grey exciton doublet.

Section 3: Additional results of experimental data.

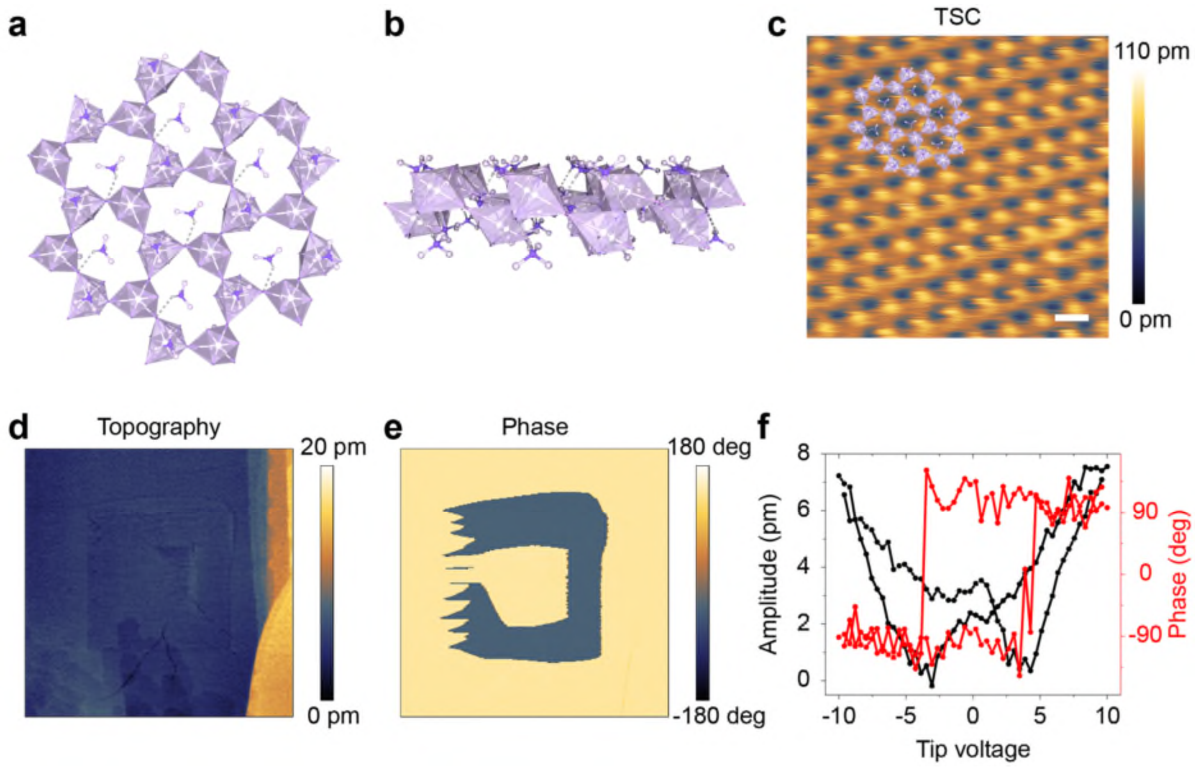

Fig. S6 | Lattice structure characterization and ferroelectric polarization switching in perovskite TSC. a, b, Top and side views of the 2D ferroelectric perovskite crystal $(TMA)_3Sb_2Cl_9$ (TSC). c, Atomic-scale ncAFM image of an as-grown TSC crystal surface, overlaid with the TSC lattice. Scale bar is 1 nm. d, e, Topography and lateral PFM phase images of a TSC crystal with opposite remnant domain configurations. f, Phase and amplitude switching spectroscopy loops as a function of tip voltage. Both hysteresis loops and switchable polarization domains demonstrates the ferroelectricity in TSC crystal.

Methods for PFM characterization. The ferroelectric domains of TSC perovskite are imaged with Piezoresponse-Lateral mode using a Bruker Dimension Icon SPM inside a glovebox (oxygen and moisture levels below 0.1 parts per million). Generally, the AC bias magnitudes were less than 500 mV, and the single resonance frequency is in the range of 750-850 kHz. We used platinum-iridium-coated, electrically conductive tips (SCM-PIT-V2) with a spring constant $k \approx 3$ N/m. During the scans, the tip force was kept around 10 nN to avoid perovskite surface damage and produce high-quality crystals.

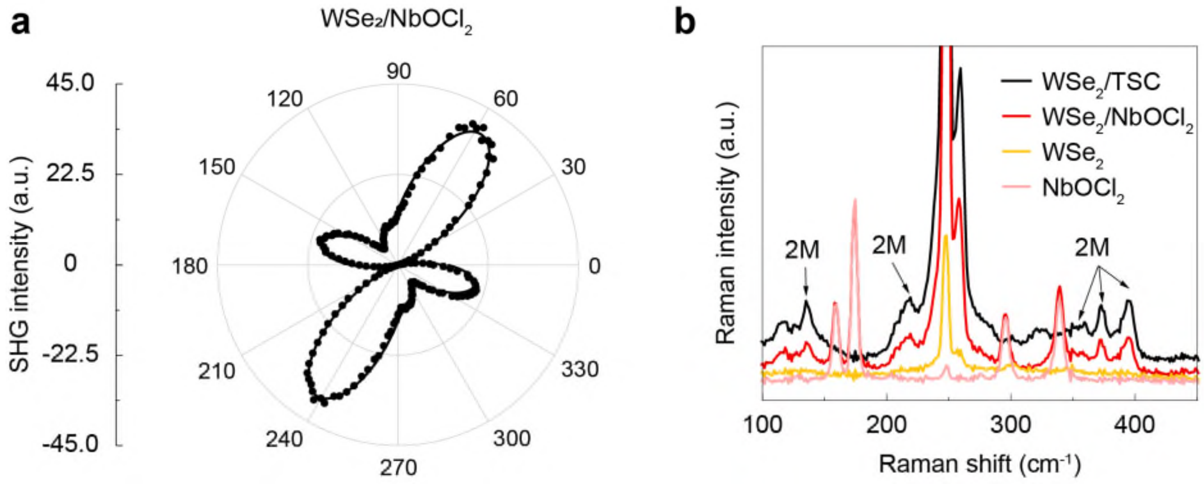

Fig. S7 | Symmetry breaking induced by the other ferroelectric material. a-b, Polar plot of the co-polarized SHG response (a) and unpolarized Raman scattering spectra (b) for a 15° stacked $WSe_2/NbOCl_2$ heterostructure, aligned along the zigzag axis of WSe_2 to the ferroelectric polarization direction of $NbOCl_2$. Here the SHG fitting is using a C_1 symmetry for WSe_2 and a C_2 symmetry for $NbOCl_2$. The observation of the same additional Raman peaks that appearance in WSe_2/TSC heterostructure suggests that: 1) the symmetry breaking occurs in WSe_2; 2) the main contributor is the in-plane electric field instead of the lattice strain caused by a certain substrate or interface geometry.

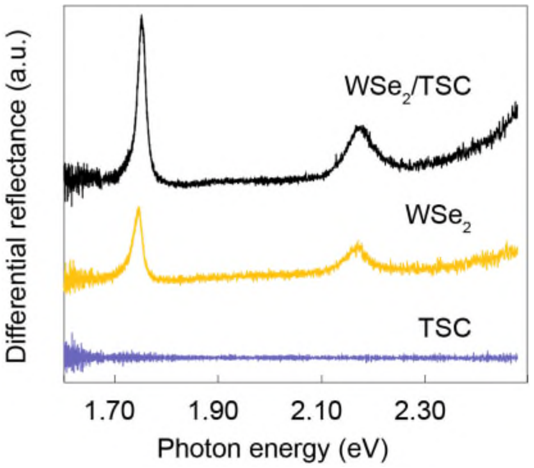

Fig. S8 | Differential-reflectance spectra. Differential-reflectance spectra of TSC perovskite, WSe_2 monolayer, and WSe_2/TSC heterostructure.

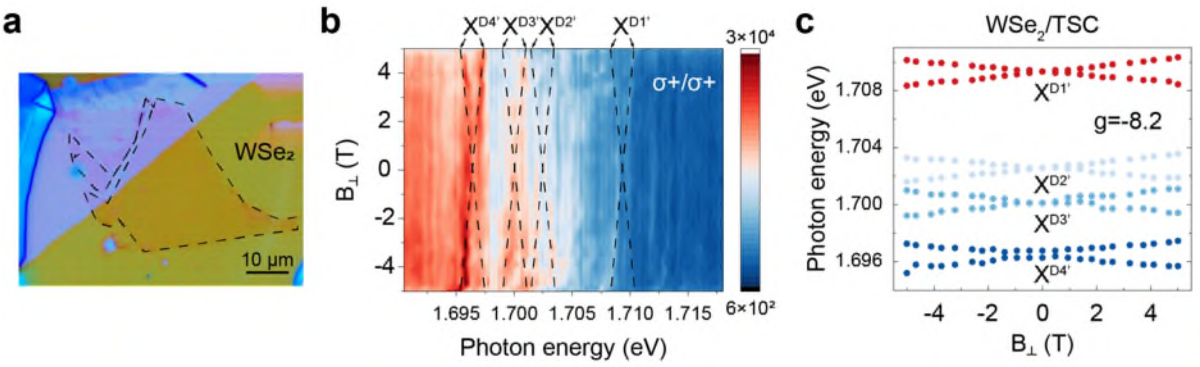

Fig. S9 | Out-of-plane magnetic-field-dependent PL spectra in a heterostructure device. a, Microscope image of a heterostructure device on Si substrate. b, Color plot of the valley-resolved PL spectra as a function of the out-of-plane magnetic field B⊥ under (σ+/ σ+) configuration. c, Zeeman splitting of each dark exciton, scaling linearly with B⊥. The fitting slope is associated with exciton g-factor, defined by $\Delta E = g\mu_B B_\perp$, where μ_B is the is the Bohr magneton. According to the prior studies[13-15], the magnitude of the g-factor is about -4 for intravalley spin-allowed neutral exciton, about -9 for intravalley spin-forbidden exciton and about -13 for intervalley momentum-forbidden exciton. The obtained g-factor of -8.2 in our measurement suggests the spin-forbidden dark nature for the emerged excitons of the heterostructure. Moreover, the obtained g-factor is a bit smaller than that of monolayer, likely due to the interband mixing, as illustrated in Supplementary Section 2.1.

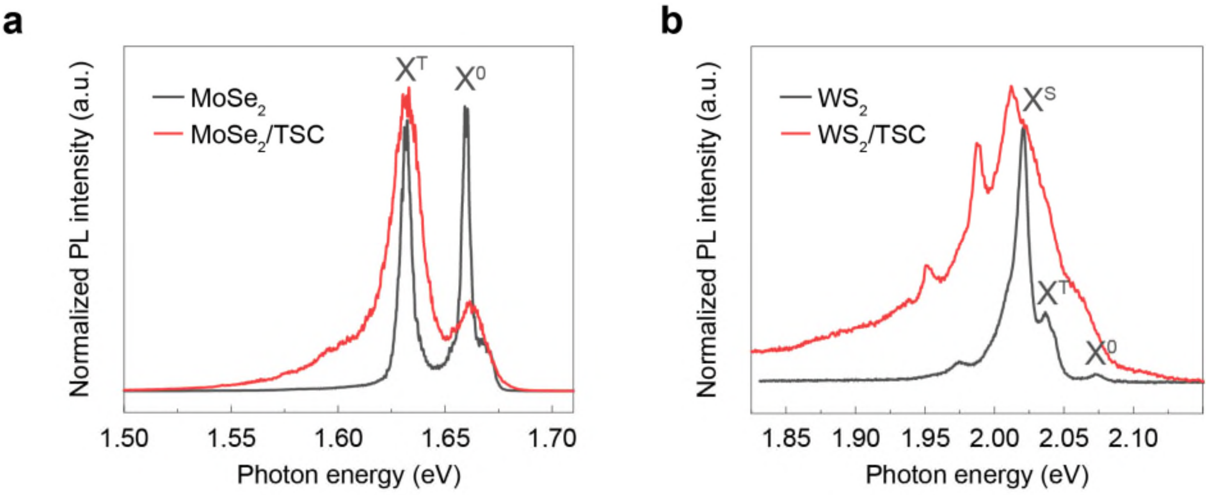

Fig. S10 | Comparison of PL spectra from heterostructures with other TMDs. a, b, PL spectra from $MoSe_2$ and WS_2 monolayers and their corresponding heterostructures.

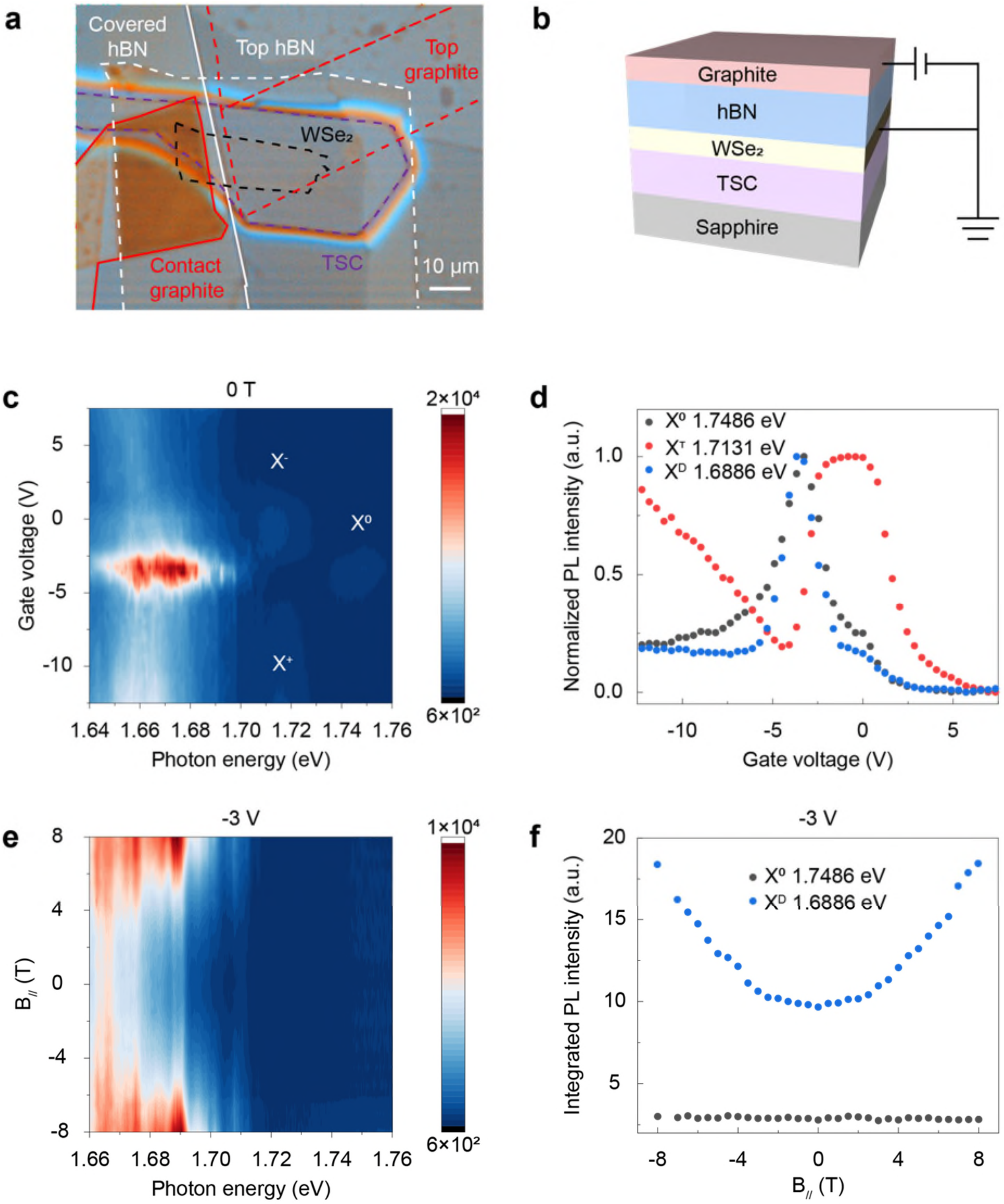

Fig. S11 | Gate dependence of the emerged dark excitons in a heterostructure device. a, Microscope image of a heterostructure device on a sapphire substrate. b, Schematic of the device, where the carrier density is controlled by a top gate. c, Gate-dependent PL map at 0 T. d, Normalized PL intensity from the neutral exciton, bright trion and brightened dark exciton as a function of gate voltage. e, Magneto-PL map at charge neutrality point (V_g = -3V). f, Integrated PL intensity from the neutral exciton and the brightened dark exciton as a function

of the in-plane magnetic field. The result from magneto-PL measurement suggests that these emerged excitonic states from the heterostructure are related to the spin-forbidden dark excitons. Moreover, based on the result from gate-dependent PL measurement, we can find that the intensities of these emerged peaks follow the gate dependence of neutral bright exciton and reach maximum at charge neutral region, indicating a charge-neutral quasiparticle nature. Therefore, we can conclude that these emerged excitons in the heterostructure have a charge-neutral and spin-forbidden dark nature.

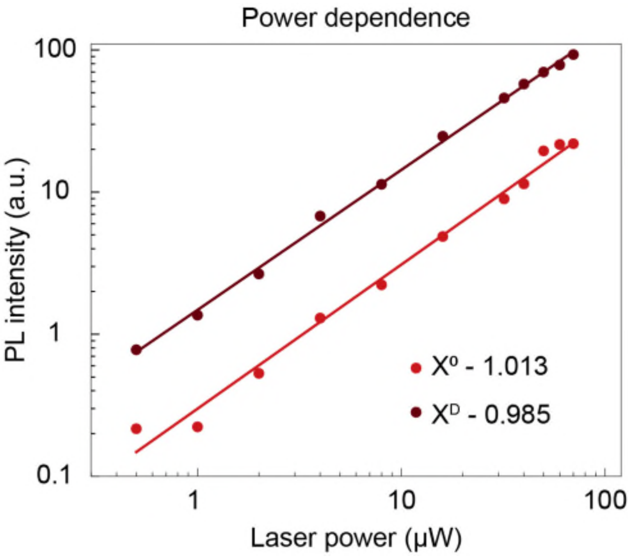

Fig. S12 | Power dependence of the dark exciton from a heterostructure. Log-plots of the power dependence of the dark (X^D) and bright (X^0) exciton. The fitting of X^D exhibits a linear power dependence with a slope of around 1, which is consistent with X^0. This power dependence rules out strongly nonlinear processes (e.g., biexciton emission). The trion assignment is excluded separately by the gate-dependent data in Fig. S11.

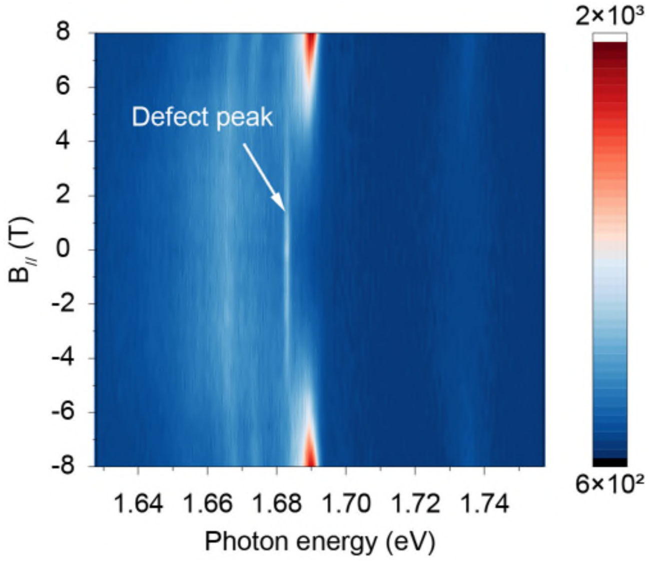

Fig. S13 | In-plane magnetic field dependence of PL spectra from a defect site in a WSe_2 monolayer. No brightening of PL emission of a defect-bounded exciton is found in a WSe_2 monolayer. Considering the localized states may also give rise to a large magnitude of g-factor due to quantum confinement effect, magneto-PL measurement with an in-plane magnetic field is employed to exclude the possibility of trap states for the emerged excitons in heterostructures.

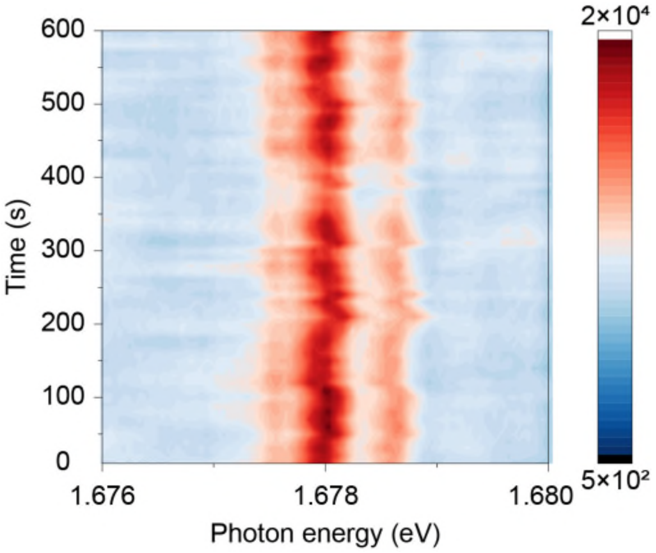

Fig. S14 | Time-trace PL emission of dark excitons. The dark-grey exciton doublet from Fig. 3e shows a correlated spectral wandering with a diffusion range of tens μeV, which possibly originates from a fluctuating inhomogeneous electric field of the underlying ferroelectric perovskite due to perturbation from the excited carriers in WSe_2.

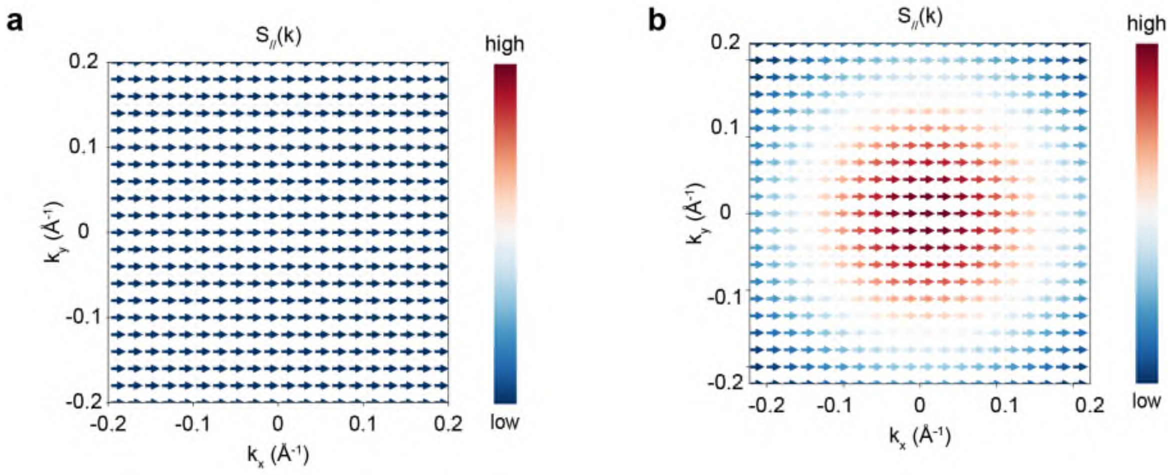

Fig. S15 | In-plane spin texture of topmost valence band. a,b, Calculated quiver maps of in-plane spin components at the highest valence band for WSe_2 in zigzag- and armchair-aligned heterostructure.

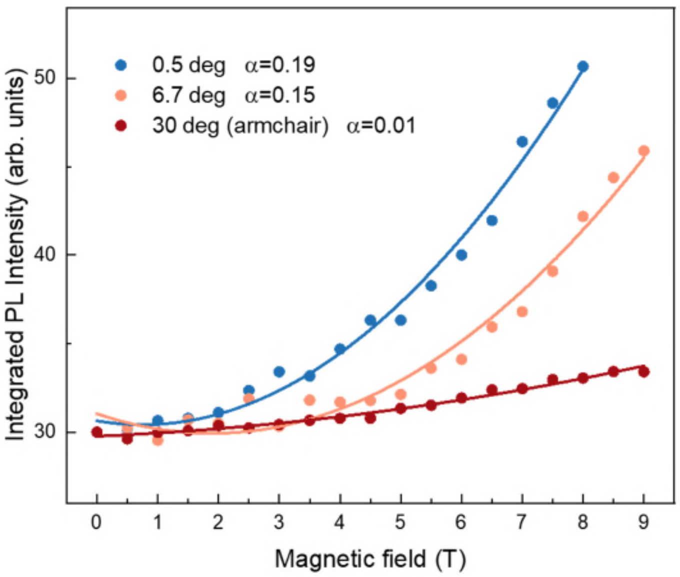

Fig. S16 | Reduced magneto-brightening curvature α with increasing twist angle.

Raman peaks (cm^{-1})	Literature mode assignment	Details: forbidden reason in strict 1st-order Raman
~150	LO_2(M)-TA(M) (~147); ZO_1(K) − TA(K) (~153)	Second-order/double-resonant feature involving zone-edge phonons (M/K). Appear when selection rules are relaxed (symmetry/disorder/resonance)[16,17].
~220	TA(M)+ZA(M) (~219) and a nearby combination TA(M)+LA(M) (~229) is commonly reported	Two-phonon combination involving M-point acoustic branches. Typically weak in pristine spectra but becomes visible with symmetry breaking/resonance[16,18,19].
~360	ZO_1(M)+TA(M) / ZO_2(K)+TA(K)	Second-order zone-edge combination (M/K). Appears as a weak band in the 350-370 cm^{-1} region. Assignments vary because multiple combination channels overlap[16].
~373–374	LO_2(M)+LA(M)	Classic second-order combination involving M-point optical and acoustic phonons. Observed when additional second-order features are activated[16,18].
~394–395	ZO_2(M)+ZA(M) (~394) and commonly reported as a high-order acoustic overtone near 395 (often labeled 3LA(M) in some papers)	High-frequency second-order zone-edge featur[19]e. Appears in the "2M/zone-edge" family and strengthens when selection rules are relaxed[16-18,20,21].

Table S1. Assignment of 2M modes.

References

1 Boyd, R. W. Nonlinear Optics, Third Edition. (Academic Press, Inc., 2008).

2 Wu, Z. et al. Intercalation-driven ferroelectric-to-ferroelastic conversion in a layered hybrid perovskite crystal. Nature Communications 13, 3104 (2022).

3 Hsu, W.-T. et al. Second Harmonic Generation from Artificially Stacked Transition Metal Dichalcogenide Twisted Bilayers. ACS Nano 8, 2951-2958 (2014).

4 Qian, Q. et al. Chirality-Dependent Second Harmonic Generation of MoS_2 Nanoscroll with Enhanced Efficiency. ACS Nano 14, 13333-13342 (2020).

5 Slobodeniuk, A. O. & Basko, D. M. Spin–flip processes and radiative decay of dark intravalley excitons in transition metal dichalcogenide monolayers. 2D Materials 3, 035009 (2016).

6 Kormányos, A., Zólyomi, V., Drummond, N. D. & Burkard, G. Spin-Orbit Coupling, Quantum Dots, and Qubits in Monolayer Transition Metal Dichalcogenides. Physical Review X 4, 011034 (2014).

7 Kormányos, A. et al. Corrigendum: k.p theory for two-dimensional transition metal dichalcogenide semiconductors (2015 2D Mater. 2 022001). 2D Materials 2, 049501 (2015).
8 Koster, G. F., Dimmock, J. O. & Wheeler, R. G. The Properties of the Thirty-Two Point Groups. (1963).
9 Glazov, M. M. et al. Spin and valley dynamics of excitons in transition metal dichalcogenide monolayers. physica status solidi (b) 252, 2349-2362 (2015).
10 Echeverry, J. P., Urbaszek, B., Amand, T., Marie, X. & Gerber, I. C. Splitting between bright and dark excitons in transition metal dichalcogenide monolayers. Physical Review B 93, 121107 (2016).
11 Robert, C. et al. Fine structure and lifetime of dark excitons in transition metal dichalcogenide monolayers. Physical Review B 96, 155423 (2017).
12 Wang, G. et al. In-Plane Propagation of Light in Transition Metal Dichalcogenide Monolayers: Optical Selection Rules. Physical Review Letters 119, 047401 (2017).
13 He, M. et al. Valley phonons and exciton complexes in a monolayer semiconductor. Nature Communications 11, 618 (2020).
14 Li, Z. et al. Momentum-Dark Intervalley Exciton in Monolayer Tungsten Diselenide Brightened via Chiral Phonon. ACS Nano 13, 14107-14113 (2019).
15 Liu, E. et al. Valley-selective chiral phonon replicas of dark excitons and trions in monolayer WSe_2. Physical Review Research 1, 032007 (2019).
16 Blaga, C. et al. Unveiling the complex phonon nature and phonon cascades in 1L to 5L WSe_2 using multiwavelength excitation Raman scattering. Nanoscale Advances 6, 4591-4603 (2024).
17 Bhatt, S. V., Deshpande, M. P., Sathe, V., Rao, R. & Chaki, S. H. Raman spectroscopic investigations on transition-metal dichalcogenides MX_2 (M = Mo, W; X = S, Se) at high pressures and low temperature. Journal of Raman Spectroscopy 45, 971-979 (2014).
18 Jeong, H. et al. Platform for surface-enhanced Raman scattering in layered quantum materials. Applied Surface Science 646, 158823 (2024).
19 Wang, X. et al. Pressure-induced iso-structural phase transition and metallization in WSe_2. Scientific Reports 7, 46694 (2017).
20 del Corro, E. et al. Excited Excitonic States in 1L, 2L, 3L, and Bulk WSe_2 Observed by Resonant Raman Spectroscopy. ACS Nano 8, 9629-9635 (2014).
21 Li, H. et al. Mechanical Exfoliation and Characterization of Single- and Few-Layer Nanosheets of WSe_2, TaS_2, and $TaSe_2$. Small 9, 1974-1981 (2013).
