# Supplementary material for: Ferroelectric brightening of spin‑forbidden dark excitons in a WSe2/hybrid-perovskite heterostructure
Source: Nat Commun. 2026 May 9;17:6285. doi: 10.1038/s41467-026-72143-y (PMC13376157; doi:10.1038/s41467-026-72143-y)
Supplement: Supplementary file 2 — Transparent Peer Review file [file 41467_2026_72143_MOESM2_ESM.pdf]

nature portfolio

Peer Review File

Ferroelectric brightening of spin-forbidden dark excitons in a WSe_2/hybrid-perovskite heterostructure

Corresponding Author: Professor Kian Ping Loh

This file contains all reviewer reports in order by version, followed by all author rebuttals in order by version.

Attachments originally included by the reviewers as part of their assessment can be found at the end of this file.

Version 0:

Reviewer comments:

Reviewer #1

(Remarks to the Author)
This manuscript reports the magnetic-field-free brightening of spin-forbidden dark excitons in monolayer WSe2 via proximity to an in-plane ferroelectric hybrid perovskite. The authors argue that the interfacial ferroelectric polarization breaks in-plane symmetry and induces an effective in-plane spin-orbit field through asymmetric inter-sublattice hopping, thereby rotating band-edge spin configurations and activating otherwise dark excitons. The effect is claimed to be twist-angle dependent and is supported by SHG symmetry analysis, magneto-PL, polarization-resolved PL, and a four-band tight-binding model.

Controlling dark excitons without magnetic fields has important applications in TMD exciton physics and valleytronics. However, there are several aspects that needs to be addressed before it can be considered further. My comments are as follows

1. As the perovskites have soft lattice, some of them show structural responses to light (e.g., photostrictive effects), meaning light can induce lattice distortions or strains. The authors should check whether, upon illumination, the TSC lattices change and whether they apply strain to the WSe2 layer.
2. In Figure 2a, it is better to plot the spectra from 1.6 to 1.70 eV, which will increase the visibility of the newly appearing peaks.
3. It would be good to show the PL data of WSe2/TSC heterostructures above the Curie temperature of TSC, where it loses in-plane ferroelectricity.
4. In Fig. S1f, the phase and amplitude data of PFM measurements require a bit more explanation. The tip voltage is applied perpendicular to the sample plane, and the ferroelectricity is claimed to be in-plane; then, what is causing this hysteresis loop? Are the dipoles get oriented out-of-plane due to the application of voltage by the PFM tip?
5. What is the typical size of the in-plane ferroelectric domain? The authors should provide PL mapping data for the heterostructures to show the uniformity of the signal.
6. If the brightening of the spin-dark states is critically dependent on the relative orientation of the layers, then the authors should pattern the TSC (like the way it was done in Figure S1e), place a WSe2 monolayer, and perform PL mapping to demonstrate how it is dependent on the in-plane polarization directions.
7. The authors should discuss why a purely orbital symmetry-breaking perturbation (without SOC-induced spin rotation) cannot explain the observations.
8. Some quantitative estimate of the interfacial electric field magnitude expected from TSC polarization is needed, and whether it is sufficient to generate the required SOC mixing should be discussed.
9. It is important to mention the effect of static in-plane electric fields from trapped charges and how that can interfere with the claim.

Reviewer #2

(Remarks to the Author)
In the manuscript entitled "Ferroelectric brightening of spin-forbidden dark excitons in a WSe2/hybrid-perovskite

heterostructure ", the authors present a method to optically brighten spin-forbidden dark excitons in monolayer WSe2 by interfacing it with an in-plane ferroelectric hybrid perovskite (TMA)3Sb2Cl9 (TSC). In this system, the ferroelectric proximity effect breaks the rotational symmetry of WSe2, inducing an asymmetric intersublattice interaction that generates an effective in-plane spin-orbit coupling (SOC) field. Overall, the manuscript is well-structured, the experimental design is novel, and the results have high scientific value and address significant challenge for controlling of dark excitons without large external magnetic fields. In conclusion, I recommend that, after addressing the following issues, this paper could be published in Nature Communications. Here are some points that the authors are suggested to pay attention to:
1.In the abstract, the authors claim that the ferroelectric proximity coupling is an "electrically reconfigurable" strategy. However, the manuscript only presents data from heterostructures with fixed twist angles (0.5° and 6.7°). The authors should either perform in-situ switching of the ferroelectric polarization to modulate the dark exciton intensity or rephrase the text to emphasize "twist-angle tunability" rather than electrical reconfigurability.
2.The theoretical model (Fig. 4f and SI Section 2) predicts that the transition dipole moment scales with |sin θ|^2, reaching a maximum at the armchair alignment (θ = 30°). However, the experimental data is limited to small angles (0.5° and 6.7°). To robustly validate the tight-binding model, data from a larger twist angle should be provided.
3.The Fig. 3e and Fig. S8 reveal significant spectral jittering of the dark exciton doublet. The authors attribute this to fluctuating electric fields from the ferroelectric substrate. This instability is detrimental to the "quantum information processing" as mentioned in the abstract. Please provide more discussion on the application limitations caused by this spectral wandering.
4.All optical measurements are at cryogenic temperatures (1.6 K / 4.2 K). A brief discussion or data showing the temperature dependence would be valuable to understand the robustness of the ferroelectric proximity effect against thermal fluctuations. As temperature increases, exciton linewidths broaden due to phonon scattering. Does the thermal broadening eventually mask the fine splitting induced by the ferroelectric proximity effect?
5.The authors briefly mention plasmonic coupling as alternative routes for brightening dark excitons (Refs. 10-12), commenting on them as requiring "sophisticated structural design". However, recent breakthroughs have successfully demonstrated room-temperature observation of dark excitons (doi.org/10.1021/acs.nanolett.5c04709; doi.org/10.1126/sciadv.aea5781) using plasmonic nanostructures. Therefore, I suggest that the authors should refer to these studies to highlight the complementary value and specific advantages of the ferroelectric strategy presented in this work.
6.There are some minor suggestions for improvement. For example, in the captions for Fig. 1d and 1e, the authors should explicitly state the color coding for the different curves. The term "pre-brightening" used in Fig. 3f to explain the reduced curvature α is somewhat colloquial and potentially confusing. It is recommended to replace it with a more physically precise term, such as "static zero-field brightening". The transition around Line 142 is abrupt, the discussion jumps from the emergence of new PL peaks directly to the optical transparency of the TSC substrate. The authors should revise this sentence to logically connect the relevant content.

Reviewer #3

(Remarks to the Author)
Please find the review report in attachment.

Version 1:

Reviewer comments:

Reviewer #1

(Remarks to the Author)
The authors have carefully addressed the concerns raised in the previous round of review and have revised the manuscript accordingly. The responses to the reviewers' comments are generally clear and satisfactory. In particular, the authors have provided additional explanations and clarifications that improve the overall clarity and rigor of the manuscript. The revisions have strengthened the presentation of the results, and the conclusions are now better supported by the data. I appreciate the authors' efforts in responding to the comments and improving the manuscript. In my opinion, the manuscript is now suitable for publication in Nature Communications in its current form.

Reviewer #2

(Remarks to the Author)
I have reviewed the revised manuscript and the authors' point-by-point responses. All of my initial questions have been clarified, and the necessary improvements have been incorporated. I have no further concerns and am pleased to recommend the paper for publication.

Reviewer #3

(Remarks to the Author)
The manuscript has been improved after the revision and my concerns are mostly addressed. I have two minor questions:
1. Comment 2: The PL spectra of WSe2 monolayer (Fig. 2a) should be plotted with the same energy scale as the heterostructure in Fig. 2b, to direct compare their difference.
2. One recent paper [Nanoscale Horizons 10, 2465-2474 (2025)] has reported the activation of dark excitons in WSe2 monolayer with the integration of an antiferromagnetic material CrCl3. This manuscript reports a similar effect, despite of

using a different ferroelectric substrate, i.e. ferroelectric hybrid perovskite. I think the authors need to better clarify the scientific innovation compared to the reference. For example, what is the difference between these two proximity effects? Is there anything new in physics or any improvement in the activation of dark excitons, in comparison to the $WSe_2/CrCl_3$ heterostructure? ...etc.

Open Access This Peer Review File is licensed under a Creative Commons Attribution 4.0 International License, which permits use, sharing, adaptation, distribution and reproduction in any medium or format, as long as you give appropriate credit to the original author(s) and the source, provide a link to the Creative Commons license, and indicate if changes were made.
In cases where reviewers are anonymous, credit should be given to 'Anonymous Referee' and the source.
The images or other third party material in this Peer Review File are included in the article's Creative Commons license, unless indicated otherwise in a credit line to the material. If material is not included in the article's Creative Commons license and your intended use is not permitted by statutory regulation or exceeds the permitted use, you will need to obtain permission directly from the copyright holder.
To view a copy of this license, visit https://creativecommons.org/licenses/by/4.0/

NCOMMS-26-004870-T

Ferroelectric brightening of spin forbidden dark excitons in a WSe_2/hybrid-perovskite heterostructure

Response Letter

Dear Reviewers,

We thank you for the careful evaluation of our manuscript and for the constructive comments. We have revised the manuscript and Supplementary Information accordingly. Below we provide a point-by-point response to all comments.

For ease of reading, reviewer comments are reproduced in blue, and our responses are in black. Key text changes are highlighted in yellow in this response letter, and all major changes are marked in red in the revised manuscript and SI. We greatly appreciate the reviewers' feedback and are glad that they found the work interesting. We have improved the manuscript based on reviewer's comments.

Responses to the reviewers' comments

Reviewer #1 (Remarks to the Author):

This manuscript reports the magnetic-field-free brightening of spin-forbidden dark excitons in monolayer WSe2 via proximity to an in-plane ferroelectric hybrid perovskite. The authors argue that the interfacial ferroelectric polarization breaks in-plane symmetry and induces an effective in-plane spin-orbit field through asymmetric inter-sublattice hopping, thereby rotating band-edge spin configurations and activating otherwise dark excitons. The effect is claimed to be twist-angle dependent and is supported by SHG symmetry analysis, magneto-PL, polarization-resolved PL, and a four-band tight-binding model. Controlling dark excitons without magnetic fields has important applications in TMD exciton physics and valleytronics. However, there are several aspects that needs to be addressed before it can be considered further.

Reply: We sincerely thank the reviewer for the careful reading of our manuscript and for the constructive and insightful comments. We are grateful for the reviewer's recognition of the significance of magnetic-field-free control of spin-forbidden dark excitons. We have revised

the manuscript and Supplementary Information to address all points raised. Below we provide a point-by-point response, and the corresponding changes have been incorporated in the revised version.

1. As the perovskites have soft lattice, some of them show structural responses to light (e.g., photostrictive effects), meaning light can induce lattice distortions or strains. The authors should check whether, upon illumination, the TSC lattices change and whether they apply strain to the WSe2 layer.

Reply: We thank the reviewer for raising this important point. We agree that photoinduced lattice responses in soft hybrid perovskites could, in principle, introduce strain and thereby influence the excitonic spectra. In our experiments, however, the excitation is tuned close to the WSe_2 bright-exciton resonance, while TSC has a large bandgap (~3 eV) and is optically transparent at the excitation and emission energies used here. Consequently, optical absorption and thus any photoinduced lattice response is expected to occur predominantly in the WSe_2 monolayer rather than in the TSC substrate.

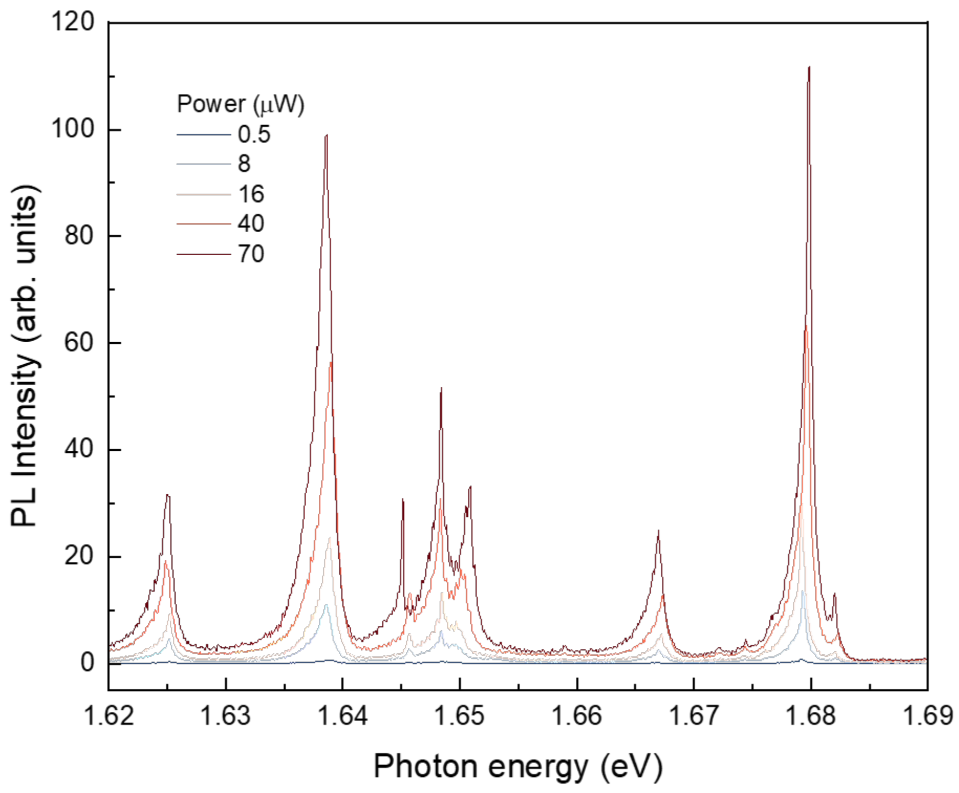

To directly assess the possibility of photoinduced strain transfer, we performed power-dependent PL measurements. Within our experimental resolution, we do not observe any systematic energy shift of the neutral exciton peak or the newly brightened low-energy features as a function of excitation power. This indicates that any photoinduced strain in TSC and the resulting strain transfer to WSe_2 are negligible under our measurement conditions. Notably, the excitation power in our measurements is low (typically ~10 μW), further minimizing photothermal or photostrictive effects.

2. In Figure 2a, it is better to plot the spectra from 1.6 to 1.70 eV, which will increase the visibility of the newly appearing peaks.

Reply: We thank the reviewer for the suggestion. We have revised Fig. 2a to better visualize the low-energy features and adjusted the plotting window at 1.55 - 1.76 eV to match the full magneto-PL map.

3. It would be good to show the PL data of WSe2/TSC heterostructures above the Curie temperature of TSC, where it loses in-plane ferroelectricity.

Reply: We previously attempted temperature-dependent PL measurements across the ferroelectric-paraelectric transition. Although the bulk crystal transitions at 380 K, we rely on thin flakes (tens of nanometers thick or thinner) for device fabrication and twist-angle identification. These thin samples degrade rapidly and irreversibly when heated near the transition temperature, preventing reliable PL measurements above the Curie point. This irreversible degradation precludes a clear comparison of PL behavior across the phase transition. To ensure that the ferroelectric proximity effect is nevertheless convincingly established, we emphasize that our main conclusions are supported by multiple independent and internally consistent observations, including (i) the twist-angle dependence, (ii) polarization-resolved PL signatures, (iii) magneto-PL behavior consistent with zero-field spin mixing, and (iv) the tight-binding model that connects the in-plane ferroelectric field to an effective in-plane SOC field.

4. In Fig. S1f, the phase and amplitude data of PFM measurements require a bit more explanation. The tip voltage is applied perpendicular to the sample plane, and the ferroelectricity is claimed to be in-plane; then, what is causing this hysteresis loop? Are the dipoles get oriented out-of-plane due to the application of voltage by the PFM tip?

Reply: We apologize that the description of the PFM measurements was too brief in the original version. In-plane ferroelectric domains produce a shear/lateral piezoelectric response. By using a lateral-PFM configuration, we can sense this in-plane signal and map domain orientation and walls. In our setup, the Bruker PFM system uses a conductive cantilever that is intentionally tilted relative to the sample surface. This tilt introduces both out-of-plane and in-plane components to the electric field applied by the tip. Although the ferroelectric polarization is primarily in-plane, the tilted tip geometry allows the electric field to couple with the in-plane polarization component. As a result, the applied voltage can effectively reorient in-plane dipoles, producing a detectable out-of-plane mechanical response via the converse piezoelectric effect.

This effect, where an in-plane polarization is probed through an out-of-plane voltage application, is a known and validated behaviour in PFM, particularly in systems with tilted cantilevers or when the ferroelectric domain structure has some out-of-plane canting (see the attached figure from Adv. Mater. 2019, 31, 1901843). These in-plane components can couple to and reverse the in-plane polarization, while the lateral PFM channel detects the corresponding in-plane piezoresponse. The observed hysteresis is therefore consistent with in-plane ferroelectricity and does not imply a permanent reorientation of dipoles to the out-of-plane direction. We have clarified this mechanism in the revised supplementary text to ensure readers understand the origin of the signal.

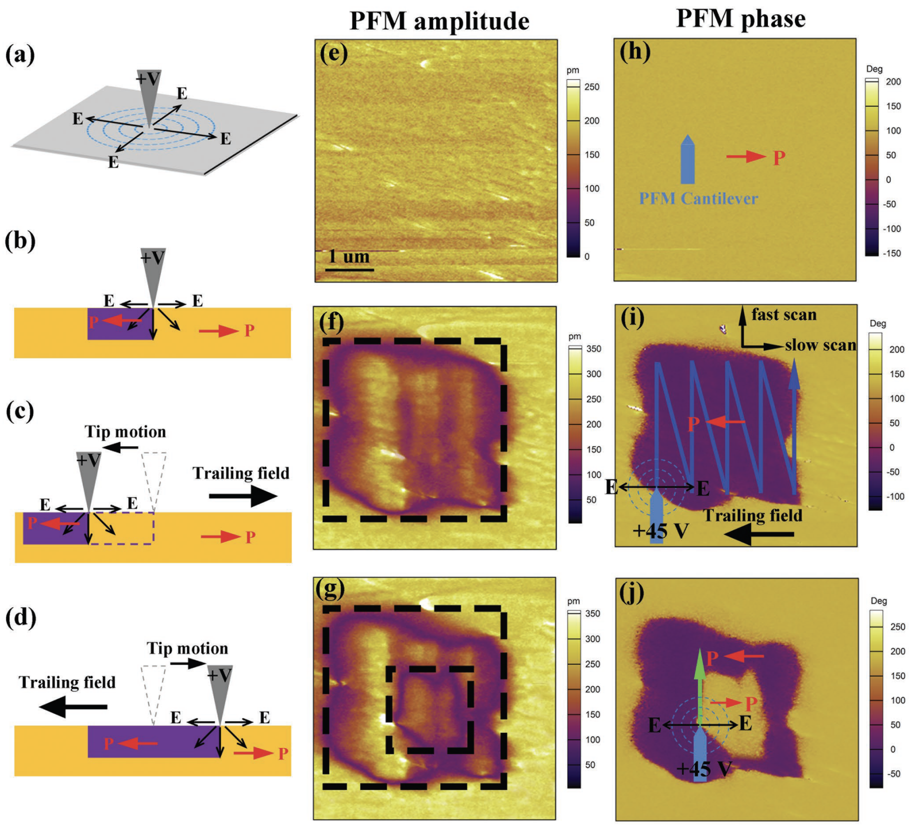

(Figure cite from Adv. Mater. 2019, 31, 1901843)

Text added in SI (Fig. S6; PFM description): Methods for PFM characterization. The ferroelectric domains of TSC perovskite are imaged with Piezoresponse-Lateral mode using a Bruker Dimension Icon SPM inside a glovebox (oxygen and moisture levels below 0.1 parts per million). Generally, the AC bias magnitudes were less than 500 mV, and the single resonance frequency is in the range of 750-850 kHz. We used platinum-iridium-coated, electrically conductive tips (SCM-PIT-V2) with a spring constant k ≈ 3 N/m. During the scans, the tip force was kept around 10 nN to avoid perovskite surface damage and produce high-quality crystals.

5. What is the typical size of the in-plane ferroelectric domain? The authors should provide PL mapping data for the heterostructures to show the uniformity of the signal.

Reply: We thank the reviewer for this suggestion and agree that both domain size and spatial uniformity should be clarified. Lateral-PFM imaging shows that TSC typically exhibits micron-scale to hundreds-of-microns-scale in-plane ferroelectric domains (depending on growth conditions). For the heterostructures studied here, we intentionally selected single-domain TSC flakes (verified by PFM prior to assembly) to avoid complications from

domain walls and mixed polarization regions. Here we show the spatial PL mapping data for representative WSe_2/TSC heterostructures. The map shows that the dark-exciton brightening is observed broadly and uniformly across the WSe_2/TSC overlap region, rather than being confined to isolated point defects or rare localized emitters.

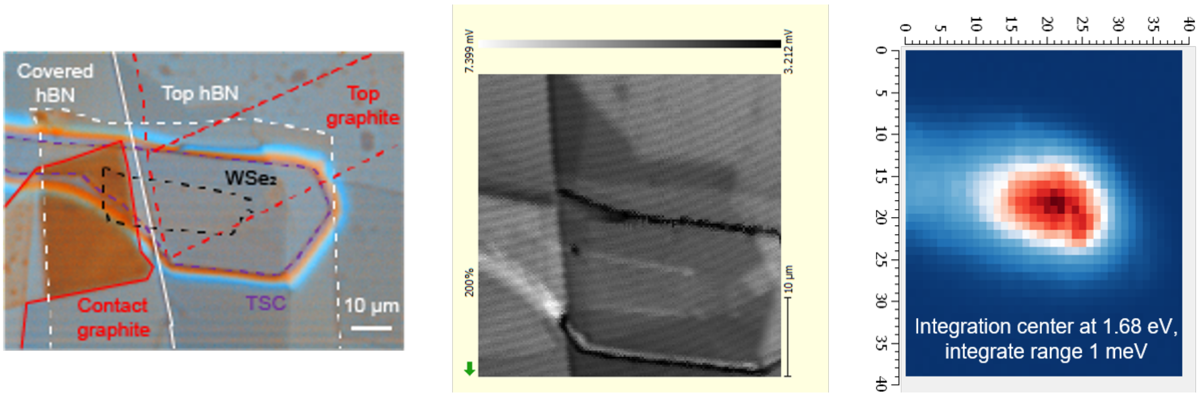

6. If the brightening of the spin-dark states is critically dependent on the relative orientation of the layers, then the authors should pattern the TSC (like the way it was done in Figure S1e), place a WSe2 monolayer, and perform PL mapping to demonstrate how it is dependent on the in-plane polarization directions.

Reply: We appreciate this constructive suggestion. In TSC, however, the in-plane polarization is crystallographically constrained to a single in-plane axis and typically forms 180° domains (+P and −P) along that axis. While PFM poling can reverse the polarization sign, it does not continuously rotate the polarization to arbitrary in-plane directions. Therefore, patterning as in Fig. S1e does not generate a set of distinct polarization angles. It only produces domains with the same in-plane axis but opposite orientation.

7. The authors should discuss why a purely orbital symmetry-breaking perturbation (without SOC-induced spin rotation) cannot explain the observations.

Reply: We thank the reviewer for highlighting this important conceptual point. The central observation in our work is the brightening of spin-forbidden dark excitons at zero magnetic field, which requires mixing between opposite-spin configurations. A purely orbital symmetry-breaking perturbation that preserves spin can relax certain optical selection rules (e.g., the momentum-forbidden intervalley excitons) and enable additional transitions within a spin-allowed manifold, but it cannot, by itself, rotate or cant the band-edge spins needed to activate spin-forbidden transitions. In contrast, an effective in-plane SOC field naturally provides the required spin mixing and also explains the finite dark-exciton emission already

present at zero magnetic field and the reduced magneto-brightening curvature at small magnetic fields due to partial static zero-field brightening. The quadratically scaling brightening under the influence of an in-plane magnetic field, demonstrated experimentally in our work, constitutes a hallmark signature of an increase in the oscillator strength of a spin dark state due to strengthened intravalley spin-mixing.

8. Some quantitative estimate of the interfacial electric field magnitude expected from TSC polarization is needed, and whether it is sufficient to generate the required SOC mixing should be discussed.

Reply: We appreciate the advice and have added more details of magnitude estimate in the main text. Actually we have discussed the magnitude at the end of section 2.3. Based on ferroelectric hysteresis measurements of TSC at room temperature, the spontaneous polarization is on the order of ~2 μC/cm^2. This corresponds to an unscreened interfacial electric field magnitude of $E = P/\varepsilon_0\varepsilon_{eff}$ ~10^9 V/m. Taking into account dielectric screening at the interface, the effective field is expected to be reduced and should remain in the ~10^8-10^9 V/m range. Within our four-band tight-binding description, the twist-angle dependent effective spin-orbit hopping energy E_{soc} can be expressed:

$$E_{soc}(\theta) = \frac{\hbar v_{diff}}{a_0} = \frac{\hbar^2 e\mathrm{E}}{a_0 m^* \Delta_0} \sin\theta$$

The calculated maximum E_{soc} is around 0.4 eV. Even allowing for substantial screening and wavefunction-overlap reduction, this estimate indicates that the ferroelectric-proximity-induced SOC is sufficiently strong to produce appreciable spin mixing and is consistent with the experimentally observed magnetic-field-free dark-exciton brightening. We have incorporated this estimate and the corresponding expression for $E_{soc}(\theta)$ in the revised manuscript.

Text added in revised manuscript: "Using the measured spontaneous polarization of around 2 μC/cm^2, we estimate an unscreened maximum magnitude for the interfacial electric field of E ~10^9 V/m. This gives rise to a spin-orbit hopping energy E_{soc} ~ 0.40 eV, which is sufficiently large to induce appreciable spin mixing."

9. It is important to mention the effect of static in-plane electric fields from trapped charges and how that can interfere with the claim.

Reply: We thank the reviewer for pointing out this relevant concern. We agree that trapped charges could generate local electrostatic fields and contribute to spectral diffusion or local symmetry breaking. However, such charge-induced fields are expected to be spatially disordered and device-dependent, and therefore would not naturally produce the systematic twist-angle dependence and the reproducible polarization behaviours observed across multiple heterostructures in our study.

Experimentally, we further mitigate and diagnose charge effects in several ways: (i) gate-dependent PL measurements are used to identify the charge-neutral regime and confirm that the newly brightened low-energy peaks track the neutral exciton rather than trion features (Supplementary Fig. S11 in revised SI) and (ii) we include control measurements on defect-localized emitters (Supplementary Fig. S13 in revised SI), which do not reproduce the same magnetic-field-free brightening behaviour.

Reviewer #2 (Remarks to the Author):

In the manuscript entitled "Ferroelectric brightening of spin-forbidden dark excitons in a WSe2/hybrid-perovskite heterostructure ", the authors present a method to optically brighten spin-forbidden dark excitons in monolayer WSe2 by interfacing it with an in-plane ferroelectric hybrid perovskite (TMA)3Sb2Cl9 (TSC). In this system, the ferroelectric proximity effect breaks the rotational symmetry of WSe2, inducing an asymmetric intersublattice interaction that generates an effective in-plane spin-orbit coupling (SOC) field. Overall, the manuscript is well-structured, the experimental design is novel, and the results have high scientific value and address significant challenge for controlling of dark excitons without large external magnetic fields. In conclusion, I recommend that, after addressing the following issues, this paper could be published in Nature Communications.

Reply: We sincerely thank the reviewer for the encouraging assessment and the constructive suggestions. We have revised the manuscript accordingly, as detailed below.

1.In the abstract, the authors claim that the ferroelectric proximity coupling is an "electrically reconfigurable" strategy. However, the manuscript only presents data from heterostructures with fixed twist angles (0.5° and 6.7°). The authors should either perform in-situ switching of the ferroelectric polarization to modulate the dark exciton intensity or rephrase the text to emphasize "twist-angle tunability" rather than electrical reconfigurability.

Reply: In the original wording, we intended to emphasize that ferroelectric polarization is switchable and can enable electrical control. However, in this manuscript we do not demonstrate in-situ polarization switching during cryogenic PL measurements. We therefore revised the abstract to avoid overstatement and to accurately emphasize the experimentally demonstrated twist-angle tunability of the ferroelectric proximity effect.

Revised abstract wording: "Our work establishes ferroelectric proximity coupling as a symmetry-tunable, magnetic-field-free strategy for controlling dark excitons in two-dimensional semiconductors."

2.The theoretical model (Fig. 4f and SI Section 2) predicts that the transition dipole moment scales with |sin θ|^2, reaching a maximum at the armchair alignment (θ = 30°). However, the experimental data is limited to small angles (0.5° and 6.7°). To robustly validate the tight-binding model, data from a larger twist angle should be provided.

Reply: We thank the reviewer for this constructive suggestion. We agree that the tight-binding model predicts a clear angular trend where the induced transition dipole strength follows $|\sin\theta|^2$, vanishing for the zigzag-aligned configuration and reaching its maximum for the armchair-aligned configuration.

Experimentally, extending the dataset to a dense set of intermediate twist angles is challenging for two main reasons. First, resolving the dark-grey exciton doublet requires exceptionally high sample quality (clean interface, minimal inhomogeneous broadening, and limited spectral wandering), which significantly constrains device yield. Second, as the twist angle increases toward the armchair alignment, the low-energy PL region becomes increasingly spectrally congested: additional proximity-induced resonances (band-splitting) emerge and partially overlap with each other. This overlap makes it difficult to unambiguously track a single pair of peaks and to extract a quantitative intensity (and hence an effective dipole strength) in a consistent manner across many angles.

To better connect experiment and theory despite these practical constraints, we have strengthened the experimental validation in the revised SI by adding magneto-PL comparison for representative devices with twist angles of 0.5°, 6.7° and 30°. We extract the magneto-brightening curvature α by fitting the dark exciton intensities to $I = I_0 + \alpha B_{\parallel}^2$. In the small perturbative regime, an in-plane magnetic field mixes the out-of-plane spin-polarized

bright and spin-forbidden dark configurations linearly in $B_{//}$, so the oscillator strength and hence the PL intensity grows approximately as $B_{\parallel}^2$. Therefore, a reduced α also provides a robust indicator of enhanced static zero-field brightening. Consistent with our tight-binding expectation that the induced dipole strength increases toward armchair alignment, we find that α decreases systematically as twist angle increases toward 30° (armchair-aligned), supporting a stronger ferroelectric-proximity-induced spin mixing at larger twist angles.

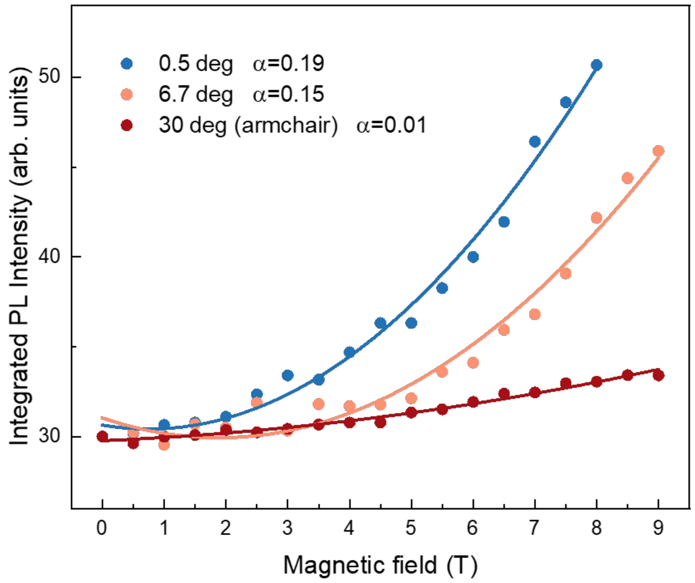

3.The Fig. 3e and Fig. S8 reveal significant spectral jittering of the dark exciton doublet. The authors attribute this to fluctuating electric fields from the ferroelectric substrate. This instability is detrimental to the "quantum information processing" as mentioned in the abstract. Please provide more discussion on the application limitations caused by this spectral wandering.

Reply: Quantum information processing requires material platforms, which host spin quasi-particles with highly tunable characteristics. In terms of excitons, long lifetimes are needed to develop protocols of coherent spin manipulation in technological timescales, e.g., via an application of appropriate trains of laser pulses. On the other hand, the efficient probing of the spin state requires emitters with high brightness for a technologically relevant exciton-to-photon spin conversion, which generally implies short exciton lifetimes. Such contrasting requirements make it paramount to develop novel strategies to activate the intrinsically dark states on demand. Achieving that via a coupling with a ferroelectric substrate exhibits technological advantages over the applications of an external magnetic field.

Our results indeed demonstrate that the ferroelectric substrate activation comes at the cost of increasing the local charge fluctuations, which translate to spectral wandering. This creates a statistical uncertainty in the determination of the energy of emission of the dark and grey excitons at a level of 200 meV. That is significantly smaller than the splitting between the grey and dark exciton states (equal to about 600 meV). Therefore, the spectral wandering does not obstruct the insight into the energetics of the system or the resolvability of individual states due to the small energy scale and correlated jittering of grey and dark exciton lines, as observable in Fig. 3e of the main text. Additionally, we found that the variations in the energy of emission do not impact the emission intensity, which is critical for the evaluation of the spin properties inspected via polarization-resolved optical spectroscopy. Therefore, for the key figures of merit used to evaluate the impact of ferroelectric substrate on dark excitons, the spectral wandering constitutes only a small contribution to the statistical uncertainty of emission energy.

For real application of quantum states (using, e.g., single photon emitters), such jittering would prevent the observability of key quantum characteristics, such as photon indistinguishability. However, we still lack the experimental demonstrations of photon indistinguishability in any type of two-dimensional material systems, while the methodology of coherent quantum manipulation of states significantly lags behind the state-of-the-art three-dimensional systems. The maturity of quantum technology in two-dimensional materials will have to increase dramatically in the future, for the level of spectral wandering observed in our experiments to become a truly meaningful bottleneck for future developments.

4.All optical measurements are at cryogenic temperatures (1.6 K / 4.2 K). A brief discussion or data showing the temperature dependence would be valuable to understand the robustness of the ferroelectric proximity effect against thermal fluctuations. As temperature increases, exciton linewidths broaden due to phonon scattering. Does the thermal broadening eventually mask the fine splitting induced by the ferroelectric proximity effect?

Reply: We thank the reviewer for this important point. We agree that temperature-dependent linewidth broadening is a key practical factor for resolving sub-meV (equal to about 600 meV) fine structure splitting between grey and dark excitons. In monolayer TMDs, the exciton PL linewidth (FWHM) is commonly described by an exciton-phonon broadening model that includes acoustic- and optical-phonon contributions:

$$\Gamma(T) = \Gamma_0 + \gamma_{ac}T + \frac{\Gamma_{0p}}{\exp\left(\frac{\hbar w_{op}}{k_B T}\right) - 1}$$

where Γ_0 is the residual (temperature-independent) linewidth (inhomogeneous broadening and radiative limit), $\gamma_{ac}T$ accounts for quasi-linear broadening from acoustic phonon scattering, and the last term captures optical-phonon-assisted broadening through the Bose-Einstein occupation factor. In our case, resolving the dark/grey fine structure requires $\Gamma(T)$ to remain comparable to or smaller than the exchange-induced splitting (ξ ~0.6 meV). Therefore, cryogenic temperatures (1.6-4.2 K) and vanishingly low inhomogeneous broadening of excitonic resonances are essential in this work to spectrally separate the two components and to quantitatively track their polarization and magneto-PL signatures. The thermal broadening of excitonic resonances constitutes a fundamental limitation for probing the dark exciton states brightened by the ferroelectric substrates exclusively at cryogenic temperatures.

Importantly, the fact that the sub-meV splitting becomes spectrally unresolved at elevated temperature does not imply that the underlying ferroelectric proximity mechanism disappears. The proximity effect is set by the interfacial ferroelectric polarization, which persists well above room temperature and only vanishes upon approaching the ferroelectric-paraelectric transition of TSC crystals (~380 K). Practically, increasing temperature mainly reduces the spectral visibility of the fine splitting and can also reduce contrast in polarization-resolved signatures via faster phonon-assisted relaxation.

5.The authors briefly mention plasmonic coupling as alternative routes for brightening dark excitons (Refs. 10-12), commenting on them as requiring "sophisticated structural design". However, recent breakthroughs have successfully demonstrated room-temperature observation of dark excitons (doi.org/10.1021/acs.nanolett.5c04709; doi.org/10.1126/sciadv.aea5781) using plasmonic nanostructures. Therefore, I suggest that the authors should refer to these studies to highlight the complementary value and specific advantages of the ferroelectric strategy presented in this work.

Reply: We sincerely thank the reviewer for pointing out these important recent advances. We have added the suggested room-temperature plasmonic studies (DOI: 10.1021/acs.nanolett.5c04709; DOI: 10.1126/sciadv.aea5781) and revised the introduction accordingly. These works clearly demonstrate that plasmonic nanostructures can enable room-temperature observation and control of dark-exciton emission by coupling to the out-of-plane dipole and accelerating radiative decay via strong Purcell enhancement.

At the same time, we now clarify that our ferroelectric proximity strategy is complementary in both mechanism and device implications. In contrast to approaches that primarily enhance emission through nanophotonic confinement, ferroelectric proximity provides a built-in, non-volatile in-plane polarization field that breaks the in-plane symmetry of WSe_2 and induces an effective in-plane SOC field, thereby directly modifying the excitonic wavefunctions/selection rules and enabling magnetic-field-free access to spin-forbidden dark excitons. Furthermore, the interlayer twist angle offers a deterministic tuning knob for the symmetry breaking and the resulting valley-coherent polarization response.

Finally, we emphasize that the plasmonic and ferroelectric strategies are not mutually exclusive: our SOC-enabled brightening mechanism can, in principle, be combined with a cavity/plasmonic platform to obtain both spin-selection-rule relaxation and additional Purcell-enhanced brightness, offering a promising route toward integrated dark-exciton photonics.

Text revised in introduction: “In this context, electric-field-assisted SOC routes are appealing in that they provide an intrinsic approach to symmetry breaking and spin mixing without external magnetic field and can be combined with optical cavities for further enhancement.”

6.There are some minor suggestions for improvement. For example, in the captions for Fig. 1d and 1e, the authors should explicitly state the color coding for the different curves. The term "pre-brightening" used in Fig. 3f to explain the reduced curvature α is somewhat colloquial and potentially confusing. It is recommended to replace it with a more physically precise term, such as "static zero-field brightening". The transition around Line 142 is abrupt, the discussion jumps from the emergence of new PL peaks directly to the optical transparency of the TSC substrate. The authors should revise this sentence to logically connect the relevant content.

Reply: We appreciate these helpful suggestions and have implemented all of them.

(i) We have revised the captions of Fig. 1d and 1e to explicitly define the color coding;

(ii) We have replaced the term “pre-brightening” with the more precise phrase “static zero-field brightening”

(iii) We have rewritten the paragraph around Fig. 2a to more smoothly connect the appearance of new low-energy PL peaks with the role of TSC as a transparent ferroelectric substrate.

Reviewer #3 (Remarks to the Author):

The manuscript entitled 'Ferroelectric brightening of spin-forbidden dark excitons in a WSe_2/hybrid-perovskite heterostructure' reports a ferroelectric proximity effect of WSe2/TSC heterostructure. The in-plane rotational symmetry of WSe2 monolayer is broken and the spin-forbidden dark excitons are brightened without a magnetic field. The authors conduct comprehensive experimental and theoretical studies, but there are still some issues remained to be solved. I can't recommend publication in Nature Communications at this stage. Below are some main concerns.

Reply: We thank the reviewer for the careful reading and for raising important concerns, especially regarding the assignment of the low-energy emission. We have substantially revised the manuscript and SI to clarify the exciton identification and to address each point as follows.

(1) In Fig. 1e, why the additional Raman peaks (noted as 2M modes) are forbidden or silent modes? Why do their emergence imply symmetry breaking? And there are multiple additional peaks with different energies, it is unclear to me that, why all of them can be associated with the symmetry breaking without considering their vibration modes and energies.

Reply: We thank the reviewer for this important question. In Fig. 1e, the additional bands labeled "2M" are not Γ-point first-order Raman modes. Instead, they correspond to finite-momentum (M/K-point) multiphonon and double-resonant Raman features that are widely reported for WSe_2, including bands near ~150 cm^{-1} (LA(M)-related), ~220 cm^{-1} (TA(M)+ZA(M) or a nearby disorder-activated zone-edge optic contribution), and a group in ~350-400 cm^{-1} (e.g., ~360, ~373-375 and ~394-395 cm^{-1}, commonly assigned to combination processes involving M-point phonons). These features are typically weak under strict selection rules because first-order Raman requires $q \approx 0$, but they can become observable and strongly enhanced under resonant conditions and when momentum conservation rules are relaxed by structural perturbations, such as strain, disorder, or interfacial symmetry lowering. To address the reviewer's request, we now explicitly assign each of the additional peaks in Fig. 1e using established literature reports of second-order modes in WSe_2 (Table R1 below), and we add the corresponding references in the revised SI.

Raman peaks (cm^{-1})	Literature mode assignment	Details: forbidden reason in strict 1st-order Raman

~150	LO_2(M)-TA(M) (~147); ZO_1(K) − TA(K) (~153)	Second-order/double-resonant feature involving zone-edge phonons (M/K). Appear when selection rules are relaxed (symmetry/disorder/resonance). (Nanoscale Advances 6, 4591-4603 (2024); Journal of Raman Spectroscopy 45, 971-979 (2014))
~220	TA(M)+ZA(M) (~219) and a nearby combination TA(M)+LA(M) (~229) is commonly reported	Two-phonon combination involving M-point acoustic branches. Typically weak in pristine spectra but becomes visible with symmetry breaking/resonance. (Nanoscale Advances 6, 4591-4603 (2024); Applied Surface Science 646, 158823 (2024); Scientific Reports 7, 46694 (2017))
~360	ZO_1(M)+TA(M) / ZO_2(K)+TA(K)	Second-order zone-edge combination (M/K). Appears as a weak band in the 350-370 cm^{-1} region. Assignments vary because multiple combination channels overlap. (Nanoscale Advances 6, 4591-4603 (2024))
~373-374	LO_2(M)+LA(M)	Classic second-order combination involving M-point optical and acoustic phonons. Observed when additional second-order features are activated. (Nanoscale Advances 6, 4591-4603 (2024); Applied Surface Science 646, 158823 (2024))
~394-395	ZO_2(M)+ZA(M) (~394) and commonly reported as a high-order acoustic overtone near 395 (often labeled 3LA(M) in some papers)	High-frequency second-order zone-edge feature. Appears in the "2M/zone-edge" family and strengthens when selection rules are relaxed. (Nanoscale Advances 6, 4591-4603 (2024); Applied Surface Science 646, 158823 (2024); Journal of Raman Spectroscopy 45, 971-979 (2014), ACS Nano, 8 (9), 9629-9635 (2014); Small 9, 1974-1981 (2013))

We have added these detailed Raman peak assignments in SI. Text in the revised manuscript:" Furthermore, the Raman spectrum of the heterostructure (Fig. 1e) exhibits several additional weak bands (2M) around ~150, ~220, and ~350-400 cm^{-1}. These features are commonly assigned to double-resonant multi-phonon Raman processes involving zone-edge (M/K) phonons21-24 (Supplementary Table S1). In an ideal crystal, first-order Raman scattering is dominated by Γ-point phonons (q≈0), so finite-momentum (M/K) contributions are typically weak. They could become observable when momentum/selection rules are relaxed by interfacial symmetry lowering, strain or disorder, consistent with our SHG evidence for

reduced symmetry. Similar 2M bands are also observed when WSe_2 is interfaced with other in-plane ferroelectrics (Supplementary Fig. S7), supporting that the symmetry reduction is imprinted into WSe_2 rather than originating from substrate vibrational modes."

(2) In Fig. 2a, PL spectra of WSe2 monolayer should also be plotted and compared. Only with the spectra of heterostructure, we don't know which features are newly emergent due to the formation of heterostructure.

Reply: We agree and have added the PL spectrum of a pristine WSe_2 monolayer in Figure 2. This makes clear which low-energy features are newly brightened in the heterostructure.

(3) 'Fig. 2a shows the PL spectra of the stacked WSe_2/TSC heterostructure, where a new group of excitonic peaks emerges'. As I understand, the authors assign the new group of excitonic peaks to spin-forbidden dark excitons, using the magneto-PL of in-plane B field. I think this claim might be wrong. First, why the spin-forbidden dark exciton is not a well-defined peak, but is a group of multiple peaks? Second, from Fig. 2a, the bright exciton energy is about 1.74 eV, the dark exciton energy is expected to be about 1.70 eV, according to the bright-dark exciton splitting (~40 meV) [Phys. Rev. Lett. 119, 047401 (2017)]. Third, I think the group of emission peaks is originated from trapped excitons that are also single photon sources [Nat. Nanotechnol. 10, 497-502 (2015)], rather than dark excitons. The g-factor is reported as 8.7, very similar as the value in this manuscript. Also, I observed similar spectra of WSe2 monolayer on a Si/SiO2 substrate at 10K, as shown below, which looks similar to Fig. 2a. I confirm that those sharp peaks manifest single photon emission. W-based TMDC monolayers, especially WSe2, have been widely reported that they have localized exciton states.

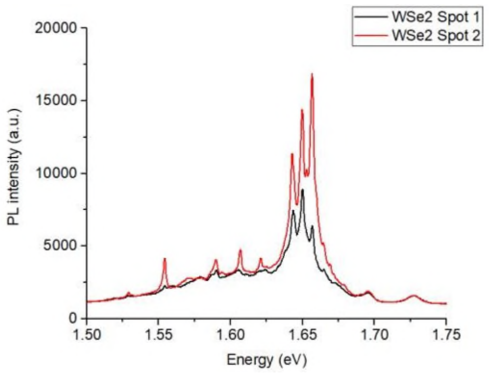

Reply: We thank the reviewer for this careful and important concern. We fully agree that monolayer WSe_2 can host defect-localized excitons (including single-photon emitters) that produce multiple sharp emission lines and may exhibit g-factors of similar magnitude.

Therefore, the g-factor alone is not sufficient for a conclusive assignment, e.g., Nature Nanotechnology 10, 497-502 (2015); Nature Nanotechnology 10, 503-506 (2015). We have revised the manuscript to clarify that our identification of the emergent low-energy features as spin-forbidden excitonic states is instead based on multiple, mutually consistent "fingerprints", and we summarize the key points here (see also our detailed response to Comment 4):

(i) Voigt-geometry magneto-PL brightening (B//): In monolayer WSe_2, the band-edge spins are predominantly Ising-like and polarized along the out-of-plane z axis. Therefore an in-plane (Voigt) magnetic field B// adds an in-plane Zeeman term that cants the electron spin away from z axis admixes a small bright-exciton component into the spin-forbidden dark/grey manifold. In the weak-field regime, the intensity increase follows an approximately quadratic dependence on B//, which is a characteristic hallmark of bright-dark spin mixing for spin-forbidden excitons in WSe_2 (Nat. Nanotechnol. 12, 883-888 (2017); 2D Mater. 4, 021003 (2017)).

(ii) Control experiment on a defect emitter: under the same Voigt-field measurement geometry, a representative defect-localized emission site in a pristine WSe_2 monolayer does not show comparable B//-induced brightening in our setup (Supplementary Fig. S13 in revised SI), helping us distinguish the emergent peaks from defect-bound emission.

(iii) Charge-neutral behaviour: gate-dependent PL shows that the relevant peaks track the neutral exciton and are maximal near charge neutrality (Supplementary Fig. S11 in revised SI), which is consistent with a neutral spin-forbidden exciton assignment.

Regarding the absolute energy position of the low-energy manifold, we note that the often-quoted bright-dark splitting of ~40 meV in monolayer WSe_2 is not a universal constant. The bright-dark separation Δ is set by the conduction-band spin-orbit splitting together with electron-hole exchange, and therefore it can be changed by the proximity-induced SOC and also vary with dielectric screening:

(i) Considering the proximity coupling: based on our four-band tight-binding description, the calculated unscreened maximum (armchair-aligned) effective spin-orbit hopping energy E_{soc} magnitude is around 0.4 eV, which could explain the red shift of the brightened dark states. Furthermore, in our WSe_2/TSC heterostructures, we additionally observe multiple satellite lines on the low-energy side, and their spectral density increases with increasing twist angle,

consistent with additional splitting/mixing of the excitonic manifold induced by the ferroelectric proximity field.

(ii) considering the dielectric screening effect: $\Delta = 40 \pm 1$ meV was reported for hBN-encapsulated WSe_2 using the in-plane light-propagation selection rules, whereas larger values around $\Delta \approx 47 - 50$ meV have been reported in more weakly screened/non-encapsulated samples (Nat. Nanotechnol. 12, 883-888 (2017); 2D Mater. 4, 021003 (2017)).

Finally, regarding why Fig. 2a (Fig. 2b,c in manuscript) shows a "group" of new exciton peaks with red shift: Fig. 2a is intended as a broad spectral overview. In the heterostructure, the low-energy window contains the canonical polarized neutral dark/grey exciton manifold together with additional satellite features. Because these weaker satellites may include proximity-induced sub-splittings and/or phonon-assisted replicas, we do not attempt to microscopically assign every weak line. Instead, our quantitative analysis focuses on the dominant and reproducible dark/grey doublet (Fig. 3), as clarified in the revised text.

Text revised in introduction: "The rich array of emergent excitonic features is likely attributable to proximity-induced band splitting and phonon-assisted processes. While a detailed microscopic assignment of each peak is beyond the scope of this work, we instead focus on elucidating the dark nature and brightening mechanism of the dominant peaks."
"To further exclude alternative assignments, we performed comprehensive gate-, power-, and magnetic-field-dependent measurements (Supplementary Figs. S11-S13). The emergent peaks maximize near the charge-neutrality region and follow the neutral-exciton gating trend, which is inconsistent with trions or other charged species. Many-body states can be excluded because their integrated intensity exhibits an approximately linear power dependence, indicative of a single-exciton transition rather than a biexciton/multiexciton complex. Finally, defect-bound emitters are ruled out because the peaks display the characteristic Voigt-geometry magnetic brightening expected for spin-forbidden excitons. In contrast, a defect-localized emitter measured under identical conditions shows no such brightening. Collectively, this evidence robustly identifies the emergent peaks as neutral, spin-forbidden excitons that are brightened via the ferroelectric proximity effect."

(4) In Fig. 3b, c, how can we assign these two peaks to dark and gray excitons? Their energies don't match the bright-dark exciton splitting, and there are much more other peaks in Fig. 2. I wonder if the whole group of emission peaks in Fig. 2 is the dark exciton, or only the assigned peaks in Fig. 3 are the dark excitons?

Reply: We thank the reviewer for raising this important point. In Fig. 3b,c we intentionally focus on the two most reproducible and spectrally resolvable low-energy peaks, which we label as the grey (X^G) and dark (X^D) components of the neutral spin-forbidden exciton manifold. This assignment does not rely on the absolute energy separation from the bright exciton alone. Instead it is based on the combined, literature-established fingerprints of the dark-grey doublet:

(i) Characteristic fine-structure splitting (X^G/X^D): The two peaks in Fig. 3 form a robust doublet with a splitting on the order of ~0.6 meV, which matches the well-known exchange-induced grey-dark splitting reported for monolayer WSe_2 (where the valley exchange interaction lifts the degeneracy and produces a "grey" component with finite z-dipole and a lower-energy "dark" component that is strictly dipole-forbidden at zero field). See for example X^G/X^D in monolayer WSe_2 from Phys. Rev. Lett. 123, 096803.

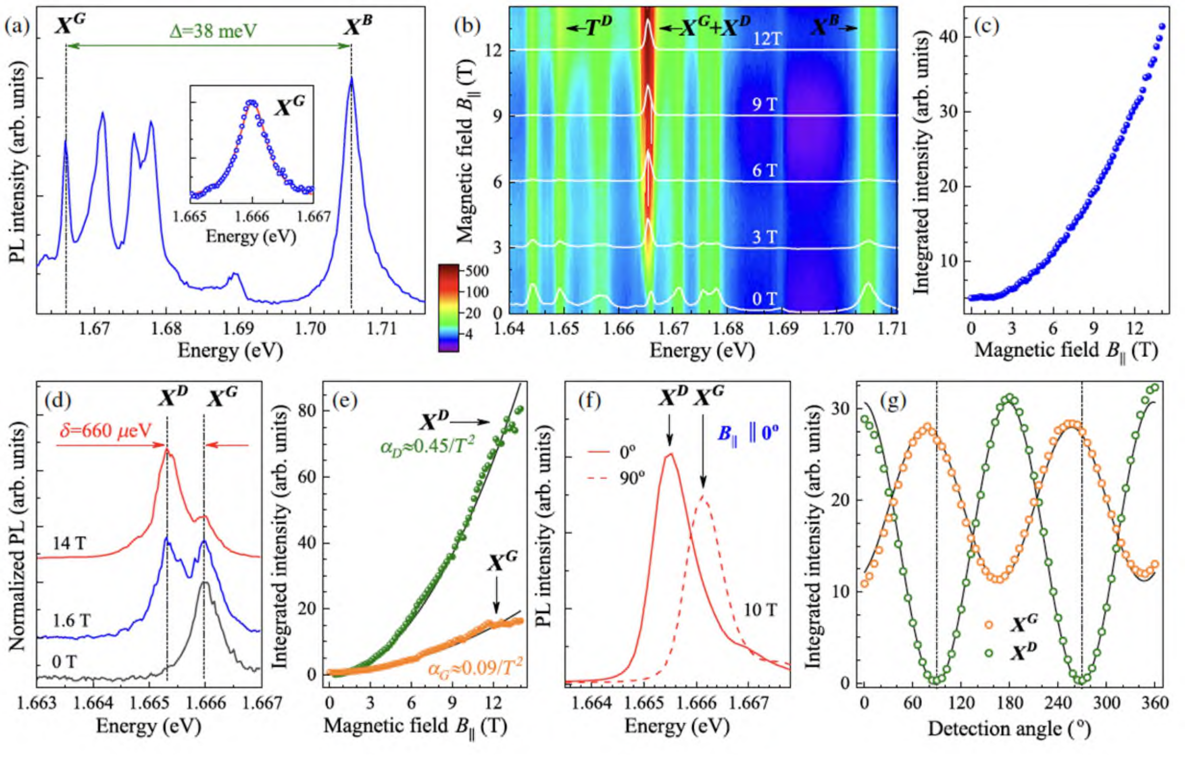

(The figure cites from Phys. Rev. Lett. 123, 096803.)

(ii) Voigt-geometry magnetic brightening consistent with spin-forbidden excitons: both peaks exhibit the characteristic B//-induced brightening behaviour (approximately $I \propto B_{//}^{2}$ in the weak-field regime), as expected when an in-plane magnetic field mixes bright and spin-forbidden exciton configurations (Nat. Nanotechnol. 12, 883-888 (2017); 2D Mater. 4, 021003 (2017)). In our heterostructure, a finite intensity already at $B_{//} = 0$ (static zero-field brightening) is additionally present, consistent with an extra spin-mixing channel from ferroelectric proximity.

(iii) Polarization behavior consistent with valley-coherent dark/grey eigenstates: The dark/grey exciton eigenstates are valley-mixed (valley-coherent) superpositions. Once they acquire brightness, they emit linearly polarized light whose polarization properties are directly connected to the valley composition and exchange-split eigenbasis. This behavior (linear polarization as a probe of the dark/grey valley eigenstates) has been established in prior polarization-resolved studies of the dark-grey doublet, e.g., Physical Review Letters 123, 096803 (2019). In our case, the two peaks exhibit distinct linear-polarization characteristics that can be systematically tracked, supporting their assignment as the canonical X^G/X^D pair.

The additional weaker satellites in Fig. 2b,c are not required for our main conclusions and are not over-interpreted. They likely reflect additional proximity-induced splitting/mixing and/or phonon-assisted processes. Because the microscopic assignment of every weak satellite peak is beyond the scope of this work, we focus our quantitative analysis on the dominant dark/grey doublet that consistently shows the established fine-structure splitting and the characteristic B//-induced brightening behavior.

(5) Is Fig. 3c and 3e plotted using the same dataset as Fig. 2b? In Fig. 2b, for the negative B field, why the PL intensity is not monotonically increasing from 0 T to -8T? For example, an emission peak close to 1.68 eV has a maximal at -7 T.

Reply: Thank you for pointing this out. Fig. 2b (Fig. 2b,c in revised manuscript) is a wide-range magneto-PL map used to illustrate the overall emergence and field response of low-energy features, whereas Fig. 3e is a higher-resolution magneto-PL map focusing on the X^D/X^G doublet (taken under higher grating). Regarding the apparent non-monotonic behaviour at a fixed photon energy in Fig. 2b: this could arise from spectral wandering of the X^D/X^G doublet, which shifts spectral weight in/out of a fixed energy bin.

(6) In Fig. 3, the linewidth of dark and gray excitons is less than 1 meV, which is extremely narrow. This is another reason that I think those emission peaks are single photon sources, instead of dark excitons.

Reply: We understand the concern. However, sub-meV linewidths are not unique to single-photon emitters. In high-quality WSe_2 monolayers at cryogenic temperature, the neutral dark exciton emission has been reported with linewidths on the order of ~1 meV or below, consistently with the Heisenberg uncertainty principle dictating the relationship of radiatively limited linewidth with the excitonic lifetime. See for example Phys. Rev. Lett. 123, 096803, Phys. Rev. B 96, 155423 and Nanoscale Horizons 10, 2465-2474 (2025), where reported linewidths are in the same order as ours, and assigned to dark/grey excitons. In our case, the identification is based on magneto-PL, the fine-structure splitting and polarization characteristics rather than linewidth alone. We also note that the emission we analyze is observed reproducibly over the heterostructure region (not isolated spatial spots), which is inconsistent with a single localized quantum emitter picture.

(7) I admit that a systemic twist-angle dependence study requires much more efforts, but the sample of 0.5°should be measured in comparison to the sample of 6.7°, to directly show the reliance of the brightening effect on the twist-angle. Now the phase difference, a variation induced by the twist-angle, is measured, but the final brightening effect is still unclear.

Reply: We thank the reviewer for this important suggestion. We agree that demonstrating the twist-angle dependence of the brightening itself is desirable. In practice, however, a direct comparison of absolute PL intensities between two independently fabricated devices (e.g., 0.5° vs 6.7°) is not a fully quantitative metric, because the detected PL intensity depends not only on the transition dipole moment (oscillator strength) but also on the exciton population and radiative quantum yield. Considering the small twist angle difference, these quantities can vary from device to device due to residual doping and sample quality, which can readily obscure a few-fold intrinsic change in oscillator strength.

For this reason, in the main text we emphasize twist-angle-sensitive observables that are much less affected by such extrinsic variations, in particular: (i) the polarization phase difference of the dark/grey doublet, which directly reflects the underlying valley-coherent eigenstates, and (ii) the magneto-PL curvature under in-plane field, which provides a robust indicator of the degree of static zero-field brightening (pre-brightening) via spin mixing. To address the

reviewer's request more directly, we now include a side-by-side Voigt-geometry magneto-PL comparison for representative devices with twist angles of 0.5°, 6.7° and 30°. Rather than comparing the absolute zero-field PL intensity across different devices, we extract the magneto-brightening curvature α by fitting the dark exciton intensities to $I = I_0 + \alpha B_{\parallel}^2$. In the small perturbative regime, an in-plane magnetic field mixes the out-of-plane spin-polarized bright and spin-forbidden dark configurations linearly in $B_{//}$, so the oscillator strength and hence the PL intensity grows approximately as $B_{\parallel}^2$. Therefore, a reduced α provides a more robust indicator of enhanced static zero-field brightening than the absolute PL intensity. Consistent with our tight-binding expectation that the induced dipole strength increases toward armchair alignment, we find that α decreases systematically as twist angle increases toward 30° (armchair-aligned), supporting a stronger ferroelectric-proximity-induced spin mixing at larger twist angles.

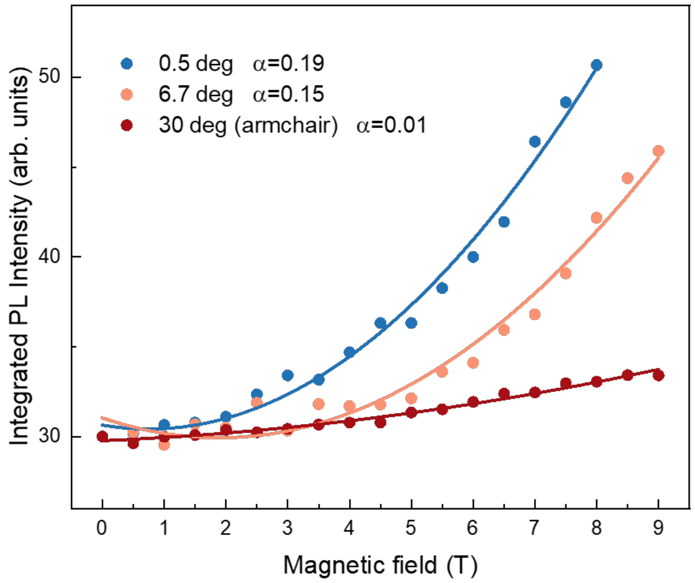

Here are some minor comments:

(1) The experiment temperatures should be indicated.

Reply: We agree and have added the measurement temperature explicitly in the captions of all relevant figures and in the Methods.

(2) The inset of Fig. 2a (reflectance spectra) can be moved to supplementary information.

Reply: We agree and moved the differential-reflectance spectra inset from Fig. 2a to the SI.

(3) ‘This material selectivity can be explained by the fact that the lowest exciton is exclusively dark in tungsten-based TMDs’. Another possible reason is that W-based TMDC monolayers have more localized excitonic states.

Reply: We appreciate this point and have revised the discussion. We clarify that our material selectivity argument is based on the ordering of bright and dark excitons, while recognizing that localized excitons may contribute additional low-energy lines in WSe_2 under certain conditions.

(4) Please unify the figure names in supplementary information. There are two name types, e.g., Fig. T1, Fig. S1.

Reply: We have renamed all figures consistently as Fig. S1, S2, … throughout the SI.

(5) ‘Consequently, any perturbation that distinguishes A from B introduces an effective intervalley-coupling term in the valley-coherent subspace’. What do A and B refer to?

Reply: We thank the reviewer for pointing out this ambiguity. In our tight-binding/Kane-Mele-type honeycomb-lattice description, A and B denote the two inequivalent basis sites within one unit cell, which is the sublattice (pseudospin) degree of freedom. Accordingly, the lattice operators in the Hamiltonian act in the {A, B} sublattice space. Therefore, a “perturbation that distinguishes A from B” refers to any term that breaks the A↔B equivalence, for example a sublattice-staggered electrostatic potential or a direction-dependent modification of the A→B vs B→A hopping amplitudes induced by the interfacial ferroelectric field. When projected onto the valley-coherent (exchange-split) exciton subspace, such a sublattice-asymmetric perturbation manifests as an effective valley-mixing (intervalley-coupling) term, which rotates the valley-coherent eigenbasis (and hence the polarization basis) of the dark/grey doublet.

In the revised manuscript, we have added a sentence to explain: “A and B denote the two inequivalent basis sites within one unit cell, which is the sublattice (pseudospin) degree of freedom”

(6) In Fig. S6, ‘This power dependence result rules out the possibility of biexciton or other more complex many-body states’. I think this is not fully correct. For example, trions can also present a linear power low.

Reply: We thank the reviewer for the correction and have revised this statement. Linear power dependence mainly rules out higher-order nonlinear processes (e.g., biexcitons) under our conditions, but does not by itself exclude trions. In our work, the trion contribution is excluded using gate-dependent measurements (Fig. S11 in revised SI). We can find that the intensities of these emerged peaks follow the gate dependence of neutral bright exciton and reach maximum at charge neutral region, indicating a charge-neutral quasiparticle nature. Moreover, the result from magneto-PL measurement suggests that these emerged excitonic states from the heterostructure are related to the spin-forbidden dark excitons. Therefore, we can conclude that these emerged excitons in the heterostructure have a charge-neutral and spin-forbidden dark nature. If these are trions, the peak intensity will increase with respective gate voltage for negative and positive trions.

We have updated the SI caption/text accordingly.

Revised SI Fig. S12 caption sentence: "This power dependence rules out strongly nonlinear processes (e.g., biexciton emission). The trion assignment is excluded separately by the gate-dependent data in Fig. S11."

NCOMMS-26-004870-A
Ferroelectric brightening of spin forbidden dark excitons in a WSe_2/hybrid-perovskite heterostructure
Response Letter (Second Revision)

Dear Reviewers,

We thank you for the further evaluation of our revised manuscript and for the constructive comments. We have revised the manuscript and Supplementary Information accordingly. Below we provide a point-by-point response to the remaining comments.

Responses to the reviewers' comments

Reviewer #1 (Remarks to the Author):

The authors have carefully addressed the concerns raised in the previous round of review and have revised the manuscript accordingly. The responses to the reviewers' comments are generally clear and satisfactory. In particular, the authors have provided additional explanations and clarifications that improve the overall clarity and rigor of the manuscript. The revisions have strengthened the presentation of the results, and the conclusions are now better supported by the data. I appreciate the authors' efforts in responding to the comments and improving the manuscript. In my opinion, the manuscript is now suitable for publication in Nature Communications in its current form.

Reply: We sincerely thank the reviewer for the positive assessment and for recognizing the improvements in clarity and rigor. We are pleased that the reviewer now finds the manuscript suitable for publication.

Reviewer #2 (Remarks to the Author):

I have reviewed the revised manuscript and the authors' point-by-point responses. All of my initial questions have been clarified, and the necessary improvements have been incorporated. I have no further concerns and am pleased to recommend the paper for publication.

Reply: We thank the reviewer for the encouraging feedback and for recommending publication. We are grateful that all questions have been clarified and that the revisions are now considered satisfactory.

Reviewer #3 (Remarks to the Author):

The manuscript has been improved after the revision and my concerns are mostly addressed. I have two minor questions:
1. Comment 2: The PL spectra of WSe2 monolayer (Fig. 2a) should be plotted with the same energy scale as the heterostructure in Fig. 2b, to direct compare their difference.

Reply: We thank the reviewer for the suggestion. We agree that using identical energy scales can, in principle, facilitate a direct visual comparison. However, the purpose of Fig. 2a is to provide a clear monolayer reference of the bright/ dark exciton region and in this spectrum the informative features are concentrated in that energy scale. Extending Fig. 2a to the broader range used in Fig. 2b would mainly add a low-energy baseline region without additional monolayer features and would compress the excitonic peaks, making their positions and linewidths less distinguishable at the figure scale. As a secondary point, we note that the monolayer spectrum was acquired using a higher grating and thus it does not extend across the full range plotted in Fig. 2b. We therefore retain the current plotting scale in Fig. 2a to maximize spectral clarity, while Fig. 2b is shown over a wider range to display the additional low-energy features that emerge in the heterostructure.

2. One recent paper [Nanoscale Horizons 10, 2465-2474 (2025)] has reported the activation of dark excitons in WSe2 monolayer with the integration of an antiferromagnetic material CrCl3. This manuscript reports a similar effect, despite of using a different ferroelectric substrate, i.e. ferroelectric hybrid perovskite. I think the authors need to better clarify the scientific innovation compared to the reference. For example, what is the difference between these two proximity effects? Is there anything new in physics or any improvement in the activation of dark excitons, in comparison to the WSe2/CrCl3 heterostructure? ...etc.
Reply: We thank the reviewer for drawing attention to this recent report (Nanoscale Horiz. 10, 2465–2474 (2025)) and agree that it is useful to clarify the distinction. We note that this work is already cited in our Introduction in the context of magnetic-field-driven brightening of dark excitons because the mechanism in $WSe_2/CrCl_3$ is a magnetic-proximity effect: the $CrCl_3$ layer provides an interfacial proximity magnetic field with an in-plane component that plays the same role as an applied in-plane magnetic field (i.e., a Zeeman-like term that mixes bright and dark configurations). In other words, although no external magnetic field is applied, the brightening remains fundamentally magnetic-field-induced through an internal exchange/proximity field from a magnetic layer.

To clarify the novelty relative to the $WSe_2/CrCl_3$ case, we summarize the key differences:

(i) Magnetic vs non-magnetic proximity. The $CrCl_3$ approach requires a magnetic material and relies on a proximity magnetic field (exchange field) to produce spin mixing. In contrast, TSC is non-magnetic, and our brightening is driven by an in-plane ferroelectric polarization at the interface.

(ii) Underlying physical mechanism. In $WSe_2/CrCl_3$, the proximity magnetic field contributes a Zeeman-like spin term. In our system, the in-plane ferroelectric polarization breaks the in-plane rotational symmetry of WSe_2 and generates a sublattice-asymmetric hopping perturbation. Combined with intrinsic spin-orbit coupling, this produces an effective in-plane spin-orbit field that cants the band-edge spins and enables static zero-field brightening of the spin-forbidden dark excitons.

(iii) Device considerations and complementarity. Magnetic-proximity approaches can be intertwined with electronic proximity (e.g., charge transfer and PL quenching) and may depend sensitively on local magnetic textures and interfacial conditions. Our ferroelectric proximity effect arises from a non-magnetic, non-volatile polarization field and is observed reproducibly across the heterostructure region, offering a complementary route that avoids introducing a magnetic layer.

We hope this detailed comparison clarifies that the present work introduces a distinct, non-magnetic proximity mechanism-ferroelectric symmetry breaking that generates an effective in-plane spin-orbit field to spin-forbidden dark excitons and that it is complementary to the magnetic-proximity strategy exemplified by $WSe_2/CrCl_3$.

Reviewer 3, attachment 1

The manuscript entitled ‘Ferroelectric brightening of spin-forbidden dark excitons in a WSe_2/hybrid-perovskite heterostructure’ reports a ferroelectric proximity effect of WSe2/TSC heterostructure. The in-plane rotational symmetry of WSe2 monolayer is broken and the spin-forbidden dark excitons are brightened without a magnetic field. The authors conduct comprehensive experimental and theoretical studies, but there are still some issues remained to be solved. I can’t recommend publication in Nature Communications at this stage. Below are some main concerns:

(1) In Fig. 1e, why the additional Raman peaks (noted as 2M modes) are forbidden or silent modes? Why do their emergence imply symmetry breaking? And there are multiple additional peaks with different energies, it is unclear to me that, why all of them can be associated with the symmetry breaking without considering their vibration modes and energies.

(2) In Fig. 2a, PL spectra of WSe2 monolayer should also be plotted and compared. Only with the spectra of heterostructure, we don’t know which features are newly emergent due to the formation of heterostructure.

(3) ‘Fig. 2a shows the PL spectra of the stacked WSe_2/TSC heterostructure, where a new group of excitonic peaks emerges’. As I understand, the authors assign the new group of excitonic peaks to spin-forbidden dark excitons, using the magneto-PL of in-plane B field. I think this claim might be wrong. First, why the spin-forbidden dark exciton is not a well-defined peak, but is a group of multiple peaks? Second, from Fig. 2a, the bright exciton energy is about 1.74 eV, the dark exciton energy is expected to be about 1.70 eV, according to the bright-dark exciton splitting (~40 meV) [Phys. Rev. Lett. 119, 047401 (2017)]. Third, I think the group of emission peaks is originated from trapped excitons that are also single photon sources [*Nat. Nanotechnol.* **10**, 497-502 (2015)], rather than dark excitons. The g-factor is reported as 8.7, very similar as the value in this manuscript. Also, I observed similar spectra of WSe2 monolayer on a Si/SiO2 substrate at 10K, as shown below, which looks similar to Fig. 2a. I confirm that those sharp peaks manifest single photon emission. W-based TMDC monolayers, especially WSe2, have been widely reported that they have localized exciton states.

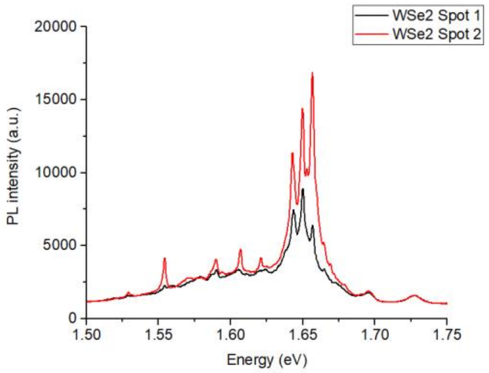

(4) In Fig. 3b, c, how can we assign these two peaks to dark and gray excitons? Their energies don't match the bright-dark exciton splitting, and there are much more other peaks in Fig. 2. I wonder if the whole group of emission peaks in Fig. 2 is the dark exciton, or only the assigned peaks in Fig. 3 are the dark excitons?

(5) Is Fig. 3c and 3e plotted using the same dataset as Fig. 2b? In Fig. 2b, for the negative B field, why the PL intensity is not monotonically increasing from 0 T to -8T? For example, an emission peak close to 1.68 eV has a maximal at -7 T.

(6) In Fig. 3, the linewidth of dark and gray excitons is less than 1 meV, which is extremely narrow. This is another reason that I think those emission peaks are single photon sources, instead of dark excitons.

(7) I admit that a systemic twist-angle dependence study requires much more efforts, but the sample of 0.5° should be measured in comparison to the sample of 6.7°, to directly show the reliance of the brightening effect on the twist-angle. Now the phase difference, a variation induced by the twist-angle, is measured, but the final brightening effect is still unclear.

Here are some minor comments:

(1) The experiment temperatures should be indicated.

(2) The inset of Fig. 2a (reflectance spectra) can be moved to supplementary information.

(3) 'This material selectivity can be explained by the fact that the lowest exciton is exclusively dark in tungsten-based TMDs'. Another possible reason is that W-based TMDC monolayers have more localized excitonic states.

(4) Please unify the figure names in supplementary information. There are two name

types, e.g., Fig. T1, Fig. S1.

(5) ‘Consequently, any perturbation that distinguishes A from B introduces an effective intervalley-coupling term in the valley-coherent subspace’. What do A and B refer to?

(6) In Fig. S6, ‘This power dependence result rules out the possibility of biexciton or other more complex many-body states’. I think this is not fully correct. For example, trions can also present a linear power low.

In summary, the authors did lots of measurements, but the analysis needs to be improved, especially the assignment of dark excitons.
